# Magnetic γFe_2_O_3_@Sh@Cu_2_O: an efficient solid-phase catalyst for reducing agent and base-free click synthesis of 1,4-disubstituted-1,2,3-triazoles

**DOI:** 10.1186/s13065-019-0657-9

**Published:** 2020-01-07

**Authors:** Fereshteh Norouzi, Shahrzad Javanshir

**Affiliations:** Heterocyclic Chemistry Research Laboratory, Department of Chemistry, Iran University of Science and Technology, Tehran, 16846-13114 Iran

**Keywords:** Click synthesis, Hybrid magnetic material, Heterogeneous catalyst, Shilajit, Humic acids

## Abstract

A hybrid magnetic material γFe_2_O_3_@Sh@cu_2_O was easily prepared from Shilajit (Sh) decorated Fe_3_O_4_ and copper acetate. The prepared magnetic hybrid material was fully characterized using different analysis, including Fourier transform infrared (FT-IR), X-ray diffraction (XRD), inductively coupled plasma (ICP), scanning electron microscopy (SEM), Energy-dispersive X-ray spectroscopy (EDX), X-ray photoelectron spectroscopy (XPS), vibrating sample magnetometer (VSM) thermal gravimetric analysis (TGA) and Brunauer–Emmett–Teller (BET). All these analysis revealed that during coating of Fe_3_O_4_@Sh using copper salt (II), synchronized redox sorption of Cu^II^ to Cu^I^ occurs at the same time as the oxidation of Fe_3_O_4_ to γFe_2_O_3_. This magnetic catalyst exhibited excellent catalytic activity for regioselective synthesis of 1,4-disubstituted-1,2,3-triazoles via one pot three-component click reaction of sodium azide, terminal alkynes and benzyl halides in the absence of any reducing agent. High yields, short reaction time, high turnover number and frequency (TON = 3.5 * 10^5^ and TOF = 1.0 * 10^6^ h^−1^ respectively), easy separation, and efficient recycling of the catalyst are the strengths of the present method.
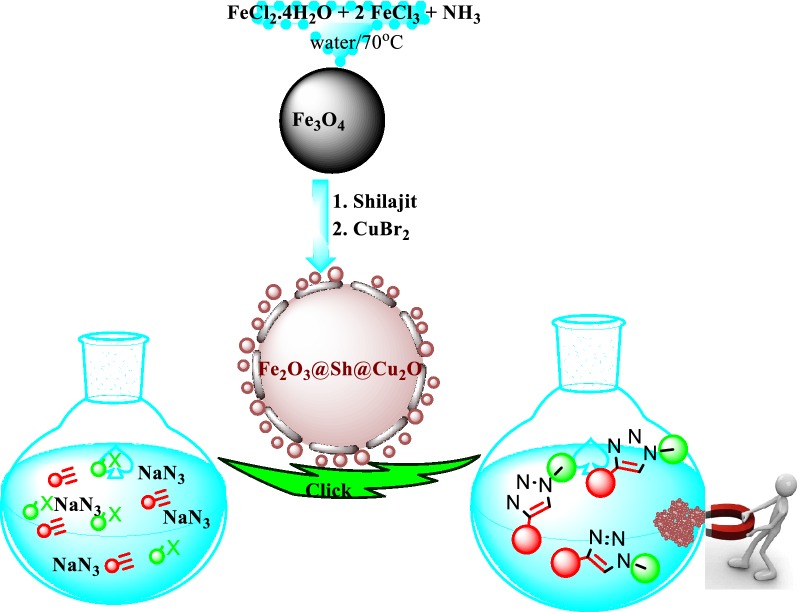

## Introduction

Presented by Sharpless [1] in 2001, the “click chemistry” consists of clipping two molecules to one another, as one closes a snap. However, not all molecules can be clipped to any other. The reaction involves an alkyne and a nitrogen-based group. For the past ten years, click chemistry has been the subject of much research. The coupling between azides and alkynes is part of the so-called bio-orthogonal chemical reactions, biocompatible reactions and a high selectivity. While click chemistry has everything to seduce the world of life, it has a weak point: its kinetics is extremely low, hence the frequent use of a catalyst, copper. The introduction of copper catalysis in 2001, independently by Meldal [2] and Sharpless groups [1] led to a major advance in both speed and regioselectivity of the reaction, where only 1,4-regioisomer is formed, and made it a reaction that respected the criteria of click chemistry.

**Fig. 1:**
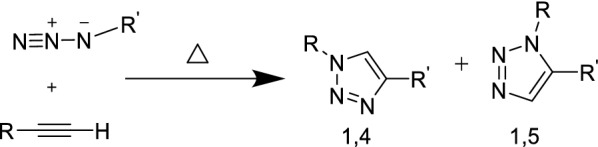
1,3-Dipolar azide/alkyne cycloaddition from Huisgen

According to the literature, several sources make it possible to obtain Cu^I^ ions in the reaction mixture. In situ reduction of copper(II) salts in the form of copper sulphate pentahydrate (CuSO_4_·5H_2_O) or copper acetate (Cu(OAc)_2_), is the most commonly encountered method. It requires the introduction of an excess reducing agent, usually sodium ascorbate. Oxidation of metallic copper is another way of generating copper(I). The reaction is done by adding a large excess of copper to the azide/alkyne mixture. Until now, the Huisgen’s copper(I)-catalyzed azide-alkyne cycloaddition (CuAAC) remain the most popular reaction making possible to rapidly, quantitatively, and reproducibly obtain a large variety of five-membered heterocycles via heteroatomic bonds [1–12]. However, the classical conditions of Huisgen reaction necessitates elevated temperatures, prolonged reaction times and lead to a mixture of isomeric 1,4- and 1,5-triazoles (Fig. 1).

**Fig. 2:**
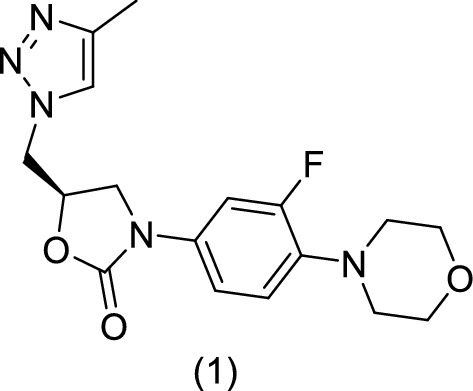
(*S*)-3-(3-Fluoro-4-morpholinophenyl)-5-((4-methyl-1*H*-1,2,3-triazol-5-yl)methyl)oxazolidin-2-one

From a biological point of view compounds comprising a triazolic group in their structures have largely aroused the attention of chemists as they present a wide range of rather potent biological activities. Demonstrating a high aromatic stability, it is resistant to acidic and basic hydrolysis, reducing and oxidative conditions and metabolic degradation. This heterocycle is therefore a good candidate for use as a modified nucleoside base [13]. Medicinal chemists have examined heterocycle synthesis based on 1,2,3-triazole as the cornerstone of medicinal chemistry and pharmaceuticals because of their important biological activities. Phillips et al. synthesized 5-(4-methyl-1,2,3-triazole)methyloxazolidinones **1** (Fig. 2) and characterized their antibacterial activity in vitro against Gram-positive and Gram-negative bacteria [14]. For example, these compounds behave as rigid binding units, so they can mimic the electronic properties of the amide bonds without the same susceptibility to hydrolytic cleavage. The 1,2,3-triazole rings have a higher dipole moment than the amide bonds, which gives them electrophilic and nucleophilic properties close to those of the peptide bonds [15].

The development of improved copper catalysts is uninterrupted. Recently, the synthesis of “click analogues” of the multivalent neoglycoconjugates was also reported using the CuAAc and organic-soluble copper catalysts [16]. Lately, Yamada et al. [17] designed an amphiphilic solid-phase self-assembled polymeric copper catalyst for click chemistry. Newly, click reaction was applied to biomolecule labeling by RIKEN institute and assembly of a biocompatible triazole-linked gene by one-pot click-DNA ligation [18]. All this research carried out by groups of researchers, elucidates not only the importance of the click reaction but also the importance of designing new catalysts that meet the demanding criteria of sustainable chemistry.

To overcome the difficulty of catalyst separation, some heterogeneous catalyst have been made such as copper(I)-modified SiO_2_ [4], nano ferrite-glutathione-copper (nano-FGT-Cu) [5], amberlyst A-21-copper(1) [6], Cu nano particles supported on agarose [7], Cu(I) on waste oyster shell powder [8], copper nanoparticles on charcoal [9], copper nanoparticles on activated carbon [1], Cu(I) supported on alumina (Cu/Al_2_O_3_) [1], copper immobilized onto a triazole functionalized magnetic nanoparticle [19], cellulose supported cuprous iodide nano particles [20], polymer supported copper [21], magnetic copper starch nanocomposite [22], knitted N-heterocyclic carbene-copper complex [23, 24], copper(I)-phosphinite complex [25], Fe_3_O_4_ nanoparticle-supported Cu(II)-β-cyclodextrin complex [26], Cu@PyIm-SBA-15 [27], Ag-Al_2_O_3_@Fe_2_O_3_ [28], and hierarchical mesoporous organic polymer Cu‐HMOP [29] for the synthesis of 1,2,3-triazoles. Despite these achievements some of these heterogeneous catalyst have significant limitations such as using reducing agent to reduce Cu(II) to Cu(I), lack of regioselectivity, by-product production, high temperature, long reaction time, and difficult conditions. So more efficient, eco-friendly, economically and simpler procedures for the synthesis of 1,2,3-triazoles are considered.

Catalysis is an essential tool of green chemistry as it enables the development of less polluting chemical processes, improvement media and opens synthetic pathway to desired products using stable resources [30]. Significant properties of catalysts are their ability to be recovered and their eco-friendly behavior. Also the majority of industrial catalysts remain heterogeneous because of the simplicity of the latter in terms of recovery and eliminating the necessity of the catalyst filtration or centrifugation after completion of the reaction [31]. Furthermore, replacement of safe organic solvent instead of hazardous organic solvent has always been a concern in green chemistry [32]. With these aspects of green chemistry in mind, we have designed and synthesized γFe_2_O_3_@Sh@Cu_2_O, a new catalysts for CuAAC reaction. Sh (mumlai in Farsi and mineral pitch in English) is a pale-brown to blackish-brown exudates obtained from layer of rocks in many mountain ranges [33–36] and it is a mixture of 85% humic acids and 15% non-humic compounds. The principle bioactive in Sh being Fulvic acid, a powerful organic electrolyte known to balance plant and animal life by increasing the electrical potential for cell restoration [36].

**Fig. 3:**
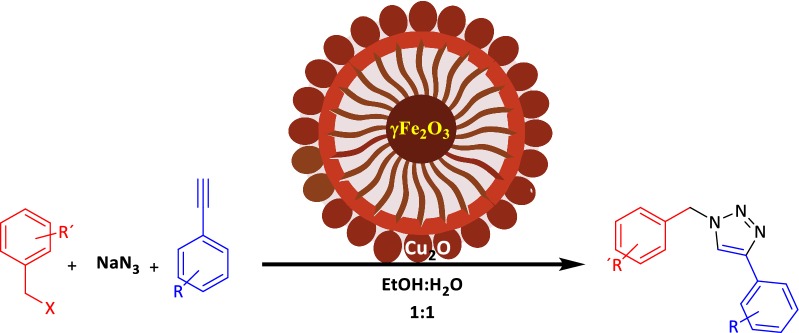
One-pot synthesis of 1,2,3-triazoles catalyzed by γ-Fe_2_O_3_@Sh@Cu_2_O

We wish to report herein the design and synthesis of a novel magnetic heterogeneous catalyst, γFe_2_O_3_@Sh@Cu_2_O, which, in minute amounts of 0.025 mol% promoted the click 1,3-dipolar cycloaddition of sodium azide, terminal alkynes and benzyl halides along with a high TOF up to 1.0 * 10^6^ (Fig. 3). γFe_2_O_3_@Sh@Cu_2_O showed good recyclability without loss of catalytic activity that could occur as a result of oxidation of the Cu(I) species, which is thermodynamically unstable, or copper leakage.

## Results and discussion

### Preparation of magnetic γFe_2_O_3_@Sh@Cu_2_O catalyst

**Fig. 4:**
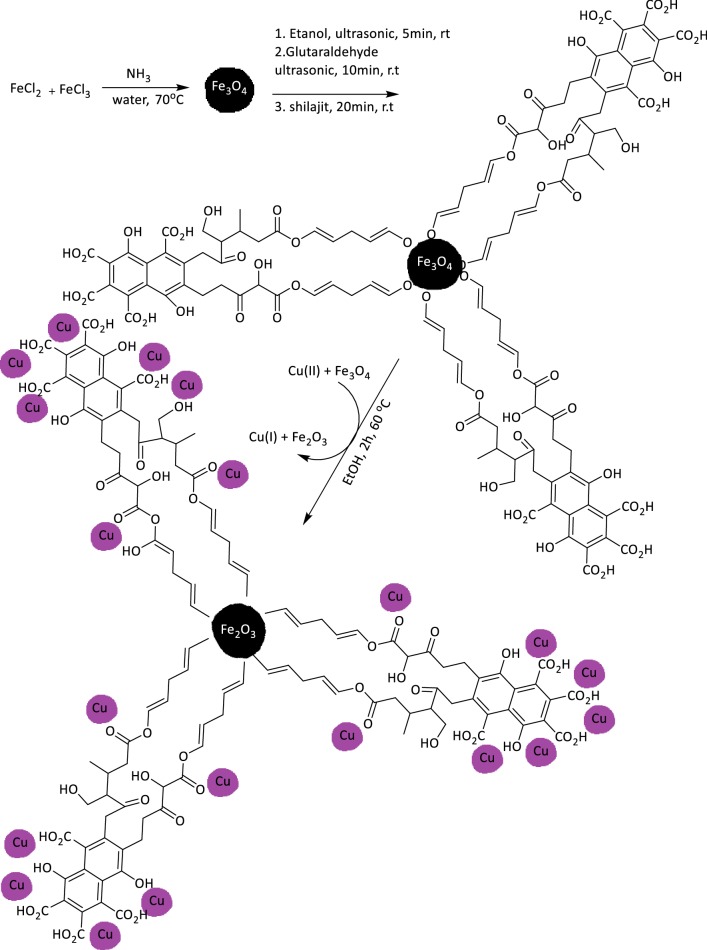
Synthesis of γFe_2_O_3_@Sh@Cu_2_O

The catalysts was prepared by a three step process (Fig. 4). First, Fe_3_O_4_ NPs were synthesized by co-precipitation method. For this purpose, FeCl_3_·6H_2_O and FeCl_2_·4H_2_O, in a 2:1 molar ratio, were dissolved in water under stirring in an inert atmosphere of nitrogen. The chemical precipitation was accomplished at 70 °C by adding a solution of ammonium (15 mL, 30 w/w). Then, the mixture of Fe_3_O_4_ and glutaraldehyde as a crosslinking agent, was sonicated in EtOH. The Sh was then added and crosslinked onto the surfaces of Fe_3_O_4_/GA NPs. Finally, CuBr_2_ and Fe_3_O_4_@Sh were clipped and thereby the hybrid magnetic catalyst was obtained after 2 h at 60 °C.

**Fig. 5:**
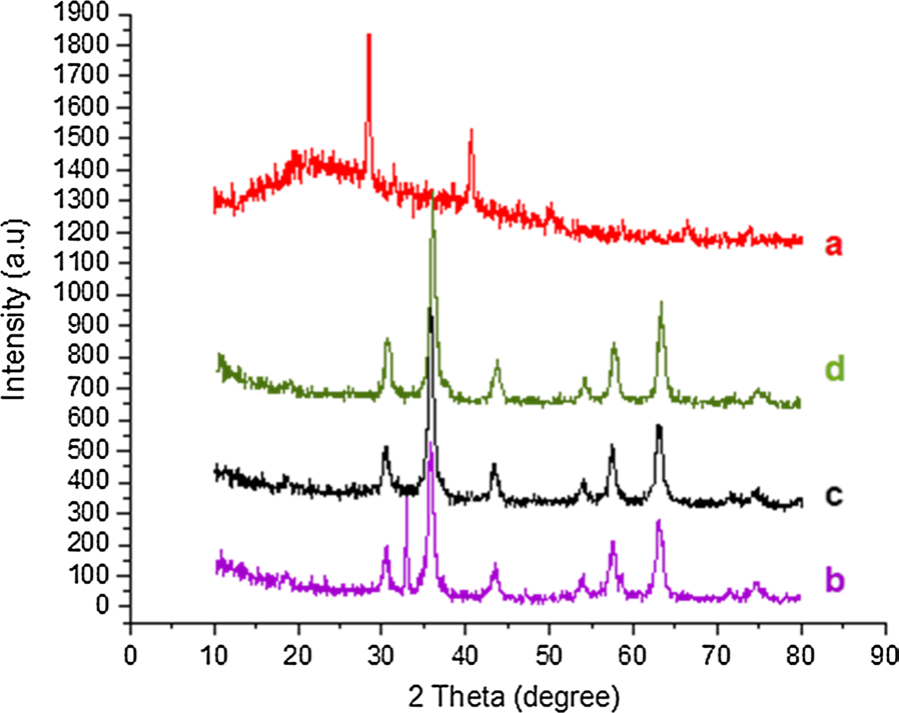
XRD pattern of (a) Shilajit, (b) Fe_3_O_4_, (c) Fe_3_O_4_@Sh, (d) γFe_2_O_3_@Sh@Cu_2_O

Characterization of prepared catalysts was performed by X-ray diffraction (XRD), Fourier transform infrared (FT-IR), field-emission scanning electron microscopy (FESEM), vibrating sample magnetometer (VSM) and X-ray photoelectron spectroscopy (*XPS*). The x-ray diffraction pattern in the 2θ range (10 to 80°) of Sh (Fig. 5a) exhibited small diffuse peaks with a few sharp peaks, implying its non-crystalline nature. XRD patterns of the prepared Fe_3_O_4_-Sh and γ-Fe_2_O_3_@Sh@Cu_2_O show that a simultaneous redox reaction has taken place, in which Cu(II) was converted to Cu(I) and Fe_3_O_4_ to γFe_2_O_3_ (Fig. 5c, d). The main diffraction peaks at 2θ = 30.1, 35.4, 43.0, 47.1, 53.4, 56.9, 62.5, 70.9, 74.9 in Fe_3_O_4_ and Fe_3_O_4_@Sh attributed to (2 2 0), (3 1 1), (4 0 0), (3 3 1), (4 4 2), (5 1 1), (4 4 0), (6 2 0), (6 2 2) crystal planes show that the Fe_3_O_4_ NPs were formed in accordance with the standard card No [01-087-2334] and the diffraction peaks at 2θ = 30.48, 33.78, 35.74, 43.69, 49.5, 54.23, 57.56, 62.73 show that the magnetite γFe_2_O_3_ NPs were formed [37] in accordance with the standard card No [01-087-2334]. As observed recycled γFe_2_O_3_@Sh@Cu_2_O retains its crystalline properties (Fig. 5e).

**Fig. 6:**
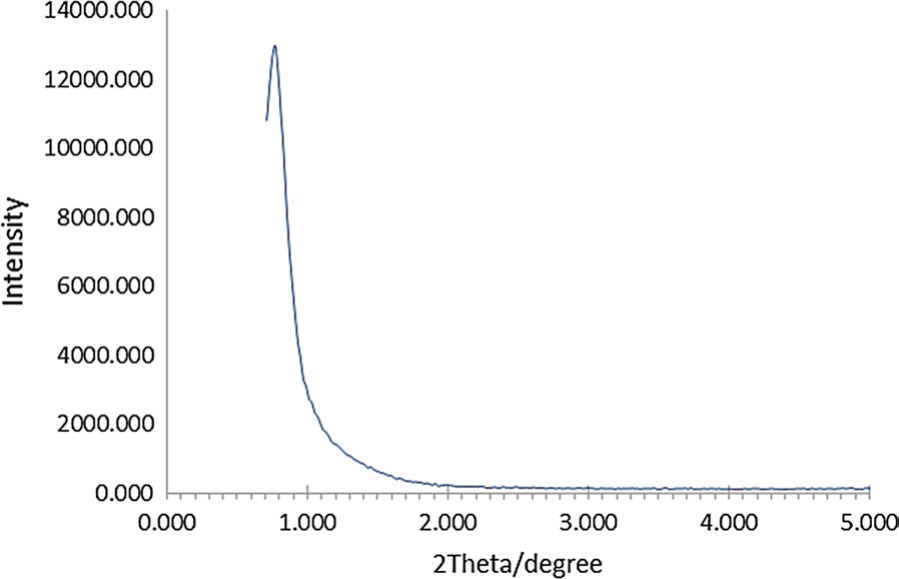
Small angle XRD pattern of γFe_2_O_3_@Sh@Cu_2_O

The average diameter of γFe_2_O_3_@Sh@Cu_2_O nanoparticle was estimated to be 25.1 nm according to the Debye–Scherrer equation ($D = k\lambda /\beta COS\theta$). The small angle XRD pattern of γFe_2_O_3_@sh@Cu_2_O is shown in Fig. 6. A broad peak at 2ϴ 0.766° was observed which is assigned to the presence of mesostructure.

**Fig. 7:**
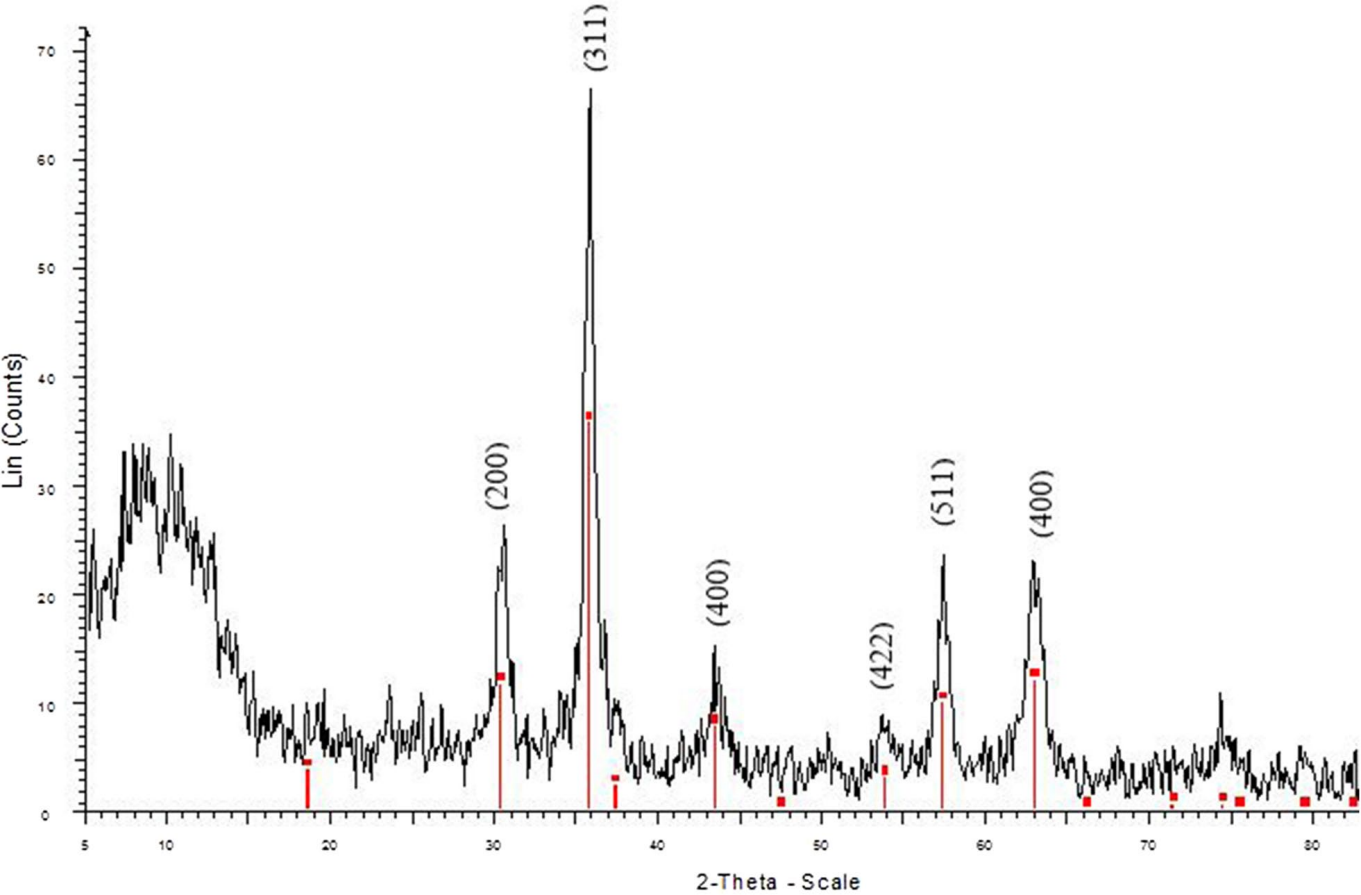
XRD pattern of recycled γFe_2_O_3_@Sh@Cu_2_O

XRD characterization of the recycled catalyst was also performed. The characteristic peaks of catalyst were still observed in recycled γFe_2_O_3_@Sh@Cu_2_O (Fig. 7) but with a significant decrease in the peak intensities. These results indicated that the structure was preserved upon after 5 run recycling; however, some collapse of the structure may have occurred (Additional file 1).

**Fig. 8:**
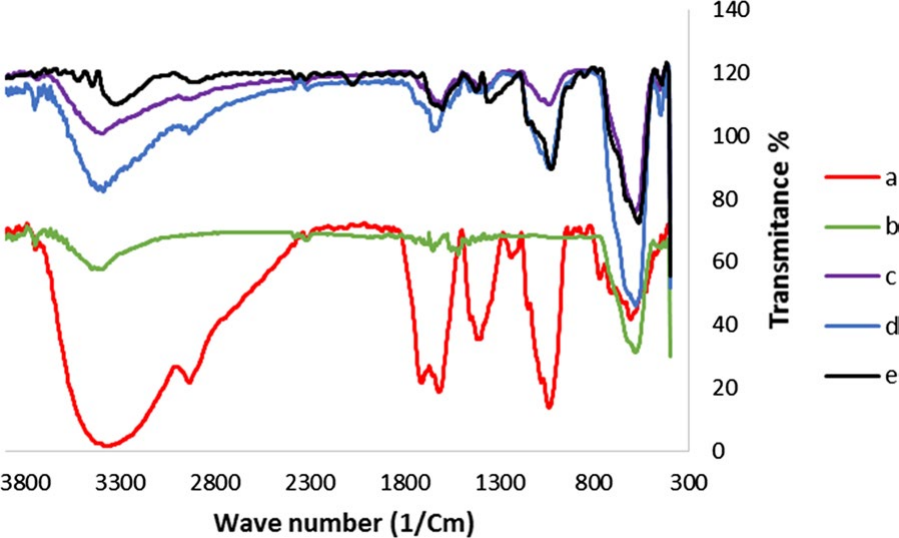
FT-IR absorption spectra for (a) Shilajit, (b) Fe_3_O_4_, (c) Fe_3_O_4_@Sh, (d) γFe_2_O_3_@Sh@Cu_2_O, (e) recycled γFe_2_O_3_@Sh@Cu_2_O after 5 times use

The FT-IR spectra of Fe_3_O_4_, Sh, Fe_3_O_4_@Sh, γFe_2_O_3_@Sh@Cu_2_O, and recycled γFe_2_O_3_@Sh@Cu_2_O after five runs are depicted in Fig. 8. The FT-IR spectrum of Sh was characterized by few broad bands at 3400, 1700 and 1650 cm^−1^ which are attributed to hydrogen bonded OH group, the stretching vibration of the carbonyl group in COOH, and C=C double bonds. Sharp bands located in the region of 2925, 1400 and 1026 cm^−1^, can be attributed to the bending vibration of aliphatic C–H groups, the O–H bending vibrations of alcohols or carboxylic acids and the OH bending deformation of carboxyl groups. For the IR spectrum of Fe_3_O_4_ the absorption band appeared at 580 cm^−1^ can be attributed to Fe–O [38]. As shown in Fig. 8, the absorption peaks in the infrared spectrum of γFe_2_O_3_@Sh@Cu_2_O at low frequencies below 600 cm^−1^ are due to Cu–O vibration [39].

**Fig. 9:**
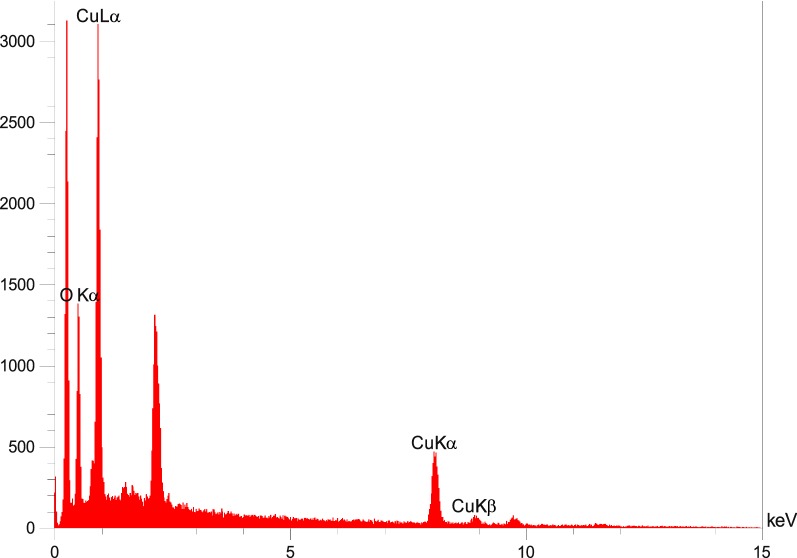
EDX spectrum of γFe_2_O_3_@Sh@Cu_2_O

Also, the EDX of γFe_2_O_3_@Sh@Cu_2_O discloses the presence of Fe, Cu, C and O in the structure of this material (Fig. 9). The copper content evaluated by ICP analysis was about 0.55%.

**Fig. 10:**
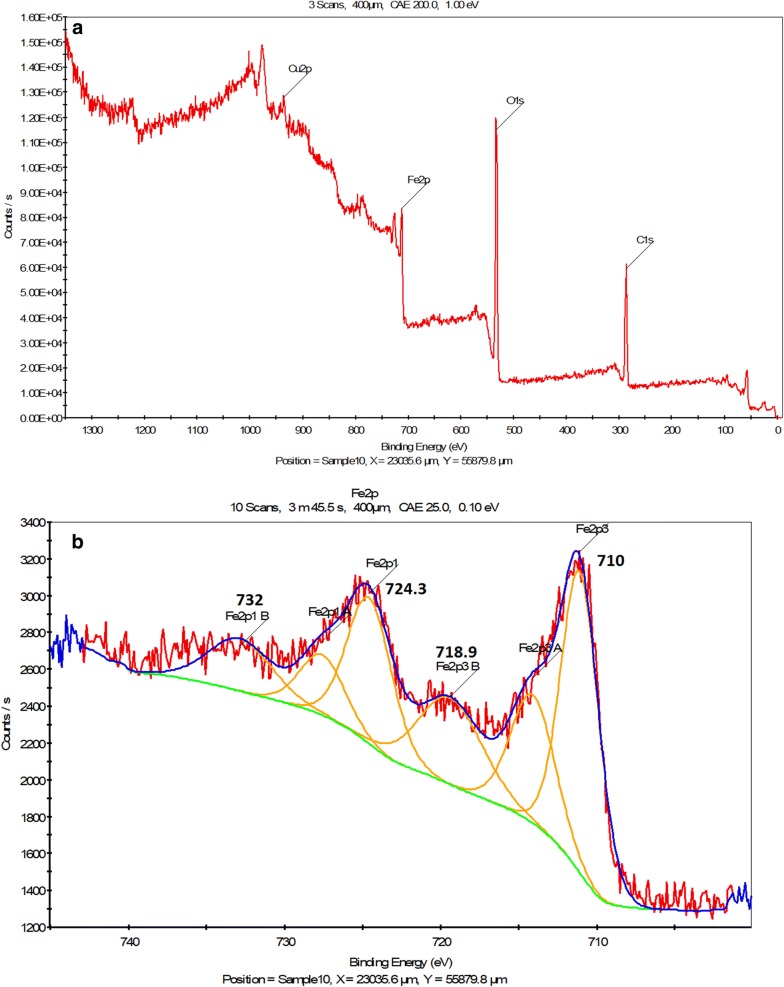

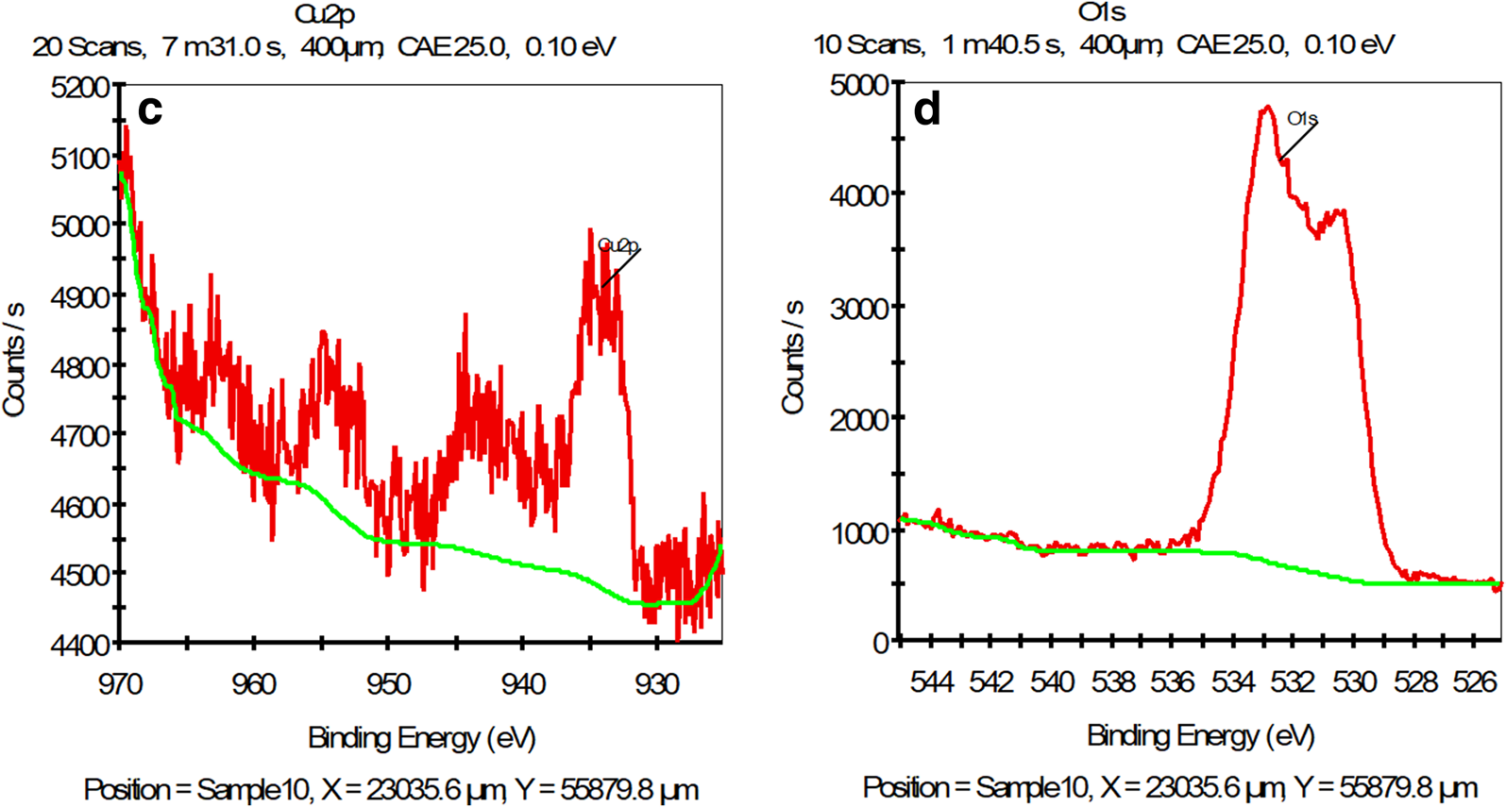
**a** XPS surface survey spectrum of γFe_2_O_3_@Sh@Cu_2_O, **b** high-resolution spectrum for the Fe2p region, **c** high-resolution spectrum for the Cu2p, **d** normalized O1s spectra

The XPS analysis of the γFe_2_O_3_@Sh@Cu_2_O nanoparticles (Fig. 10) revealed the characteristics peaks for C 1s (284.88), O1s (530.39), Fe 2p (710.89) and Cu 2p (933.01). Moreover, the high resolution narrow scan for Fe 2p in γFe_2_O_3_@Sh@Cu_2_O display energy peak of Fe2p3/2A and Fe2p1 at 710.8 and 724.3 eV respectively, which are characteristic peaks of the 3+ ion and clearly indicate the formation of the γ-Fe_2_O_3_ [40, 41]. Moreover, there exist satellite peak at 718.9 eV sides of the main doublet peaks, which also indicate the absence of the 2^+^ ion, suggesting that the Fe_3_O_4_ nanoparticles were partly oxides and CuO nanoparticles were reduced, and γFe_2_O_3_@Sh@Cu_2_O was created. The Cu_2_p3/2 peaks located at 933.0 eV was attribute to Cu^1^ in Cu_2_O. Moreover, the O1s peaks at 530.4 eV are coherent with O-state in Cu_2_O.

**Fig. 11:**
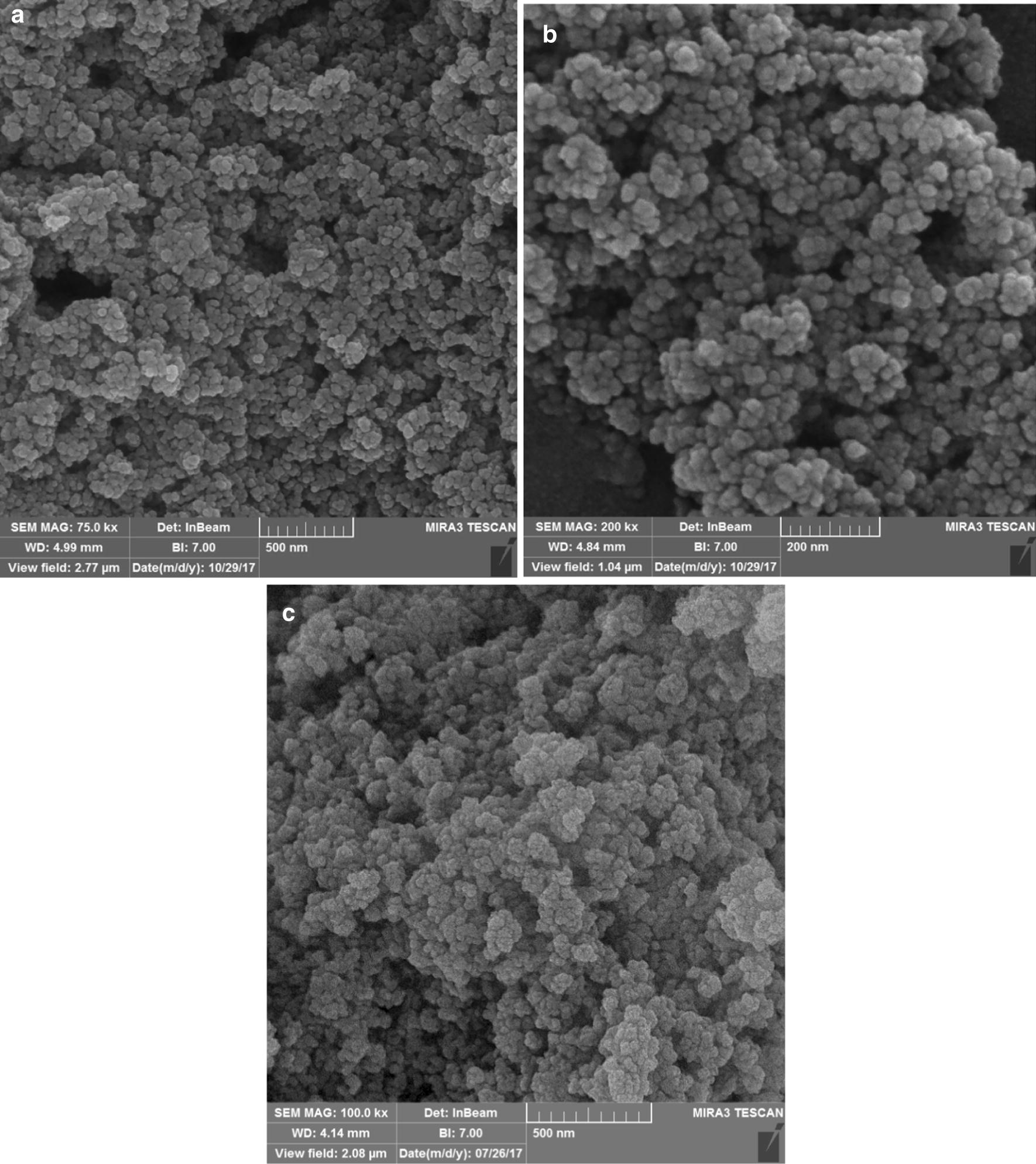
SEM image of **a** Fe_3_O_4_, **b** Fe_3_O_4_@Sh, **c** γFe_2_O_3_@Sh@Cu_2_O

The morphology and size of Fe_3_O_4_, Fe_3_O_4_@Sh and the synthesized γFe_2_O_3_@Sh@Cu_2_O NPs were investigated using SEM analysis (Fig. 11a–c). The SEM image of the synthesized γFe_2_O_3_@Sh@Cu_2_O NPs (Fig. 11c) shows that morphology of the particles is spherical or quasi-spherical and the surface configuration of NPs is quite rough with smaller subunits. The average nanoparticle diameter of γFe_2_O_3_@Sh@Cu_2_O was estimated 24–26 nm based on the SEM image.

**Fig. 12:**
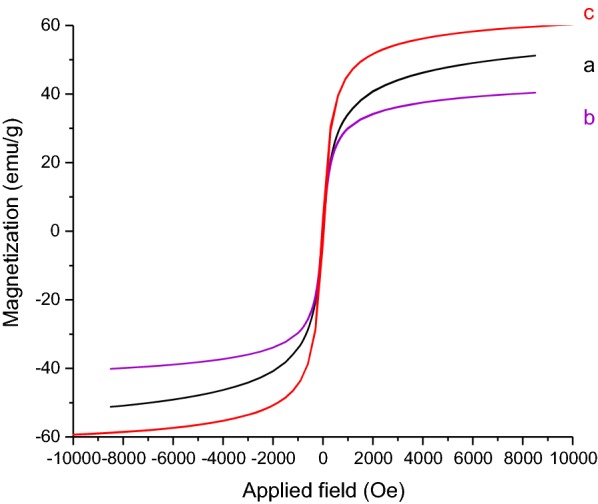
Magnetization curve of (a) Fe_3_O_4_, (b) Fe_3_O_4_@Sh, (c) γFe_2_O_3_@Sh@Cu_2_O

The magnetic properties of the prepared γFe_2_O_3_@Sh@Cu_2_O were measured by VSM at room temperature with the field sweeping from − 8500 to + 8500 oersted (Fig. 12). The magnetic curve of γFe_2_O_3_@Sh@Cu_2_O revealed that it has super magnetic behavior, and its magnetization values was found to be 58 emu g^−1^, so it could be efficiently separated by an external permanent magnet.

**Fig. 13:**
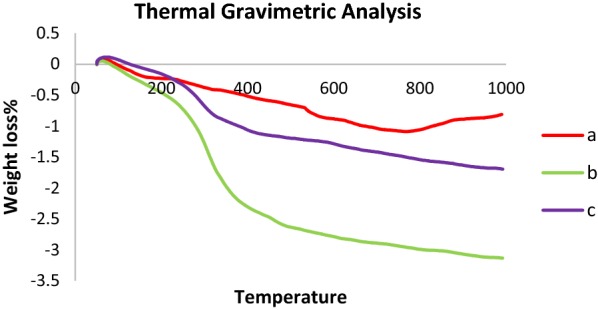
Thermal gravimetric of (a) Fe_3_O_4_, (b) Fe_3_O_4_@Sh, (c) γFe_2_O_3_@Sh@Cu_2_O

To investigate the thermal stability of catalyst, thermogravimetric analysis was carried out from 25 to 1000 °C under oxygen atmosphere condition. The TGA curves of Fe_3_O_4_, Fe_3_O_4_@Sh and γFe_2_O_3_@Sh@cu_2_O, illustrating the variations of residual masses of the samples with temperature, are shown in Fig. 13a–c. The first mass loss of, 0.3% for Fe_3_O_4_ and γFe_2_O_3_@Sh@cu_2_O and 0.6% for Fe_3_O_4_@Sh, observed below 260 °C, was attributed to moisture elimination. The total weight loss of the uncoated Fe_3_O_4_, Fe_3_O_4_@Sh and γFe_2_O_3_@Sh@cu_2_O are 1.07, 3.1 and 1.7% respectively, which revealed that the thermal stability of γFe_2_O_3_@Sh was enhanced prominently after coating with Cu_2_O.

**Fig. 14:**
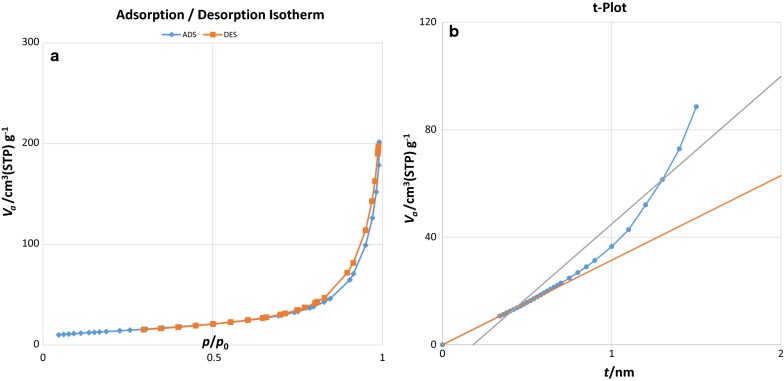
**a** N_2_ adsorption/desorption isotherms and **b** t-plot for γFe_2_O_3_@Sh@Cu_2_O

The surface area and pore volume of γ-Fe_2_O_3_@Sh@Cu_2_O were estimated from the N_2_ adsorption/desorption isotherms and T-plot (Fig. 14a, b). Vertical plots from the straight line in T-plot indicated the presence of mesopores [42]. Applying the Barrett-Joyner-Halenda (BJH) method, indicates that the sample contains mesopores with diameters close to 23.655 nm and a surface area of 49.746 m^2^/g.

Considering the efficiency of γFe_2_O_3_@Sh@Cu_2_O, the reaction of benzyl chloride, sodium azide and phenyl acetylene approved as a model reaction. The impact of different parameters such as kinds and amounts of catalysts, solvents, time, and temperature reaction was checked to obtain the best combination condition. When the reaction was attempted without a catalyst in water, without water, at room temperature, at 80 °C that product was not obtained even after 48 h (Table 1, entry 1–4). To optimize the reaction conditions, several green solvents were used in different proportions. The effect of different solvents on the reaction efficiency is summarized in the Table 1. From Table 1, it was found that H_2_O:EtOH (1:1) was the most effective solvent, while the use of other solvents such as EtOH and other proportions of H_2_O:EtOH resulted in lower yields.

**Table 1:** Optimize the three-component reactions condition of benzyl bromide, sodium azide and phenyl acetylene

Entry	Catalyst (mg)	Solvent	Temperature	Time	Isolated yield %
1	–	–	r.t	48 h	–
2	–	H_2_O	r.t	48 h	–
3	–	H_2_O	80 °C	48 h	–
4	–	–	80 °C	48 h	–
5	5	H_2_O	80 °C	1 h	57
6	5	H_2_O:EtOH (1:1)	80 °C	1 h	80
7	5	H_2_O:EtOH (1:2)	80 °C	1 h	70
8	5	H_2_O:EtOH (2:1)	80 °C	1 h	63
9	5	EtOH	80 °C	1 h	74
10	5	H_2_O:EtOH (1:1)	80 °C	45 min	83
11	5	H_2_O:EtOH (1:1)	80 °C	2 h	60
12	5	H_2_O:EtOH (1:1)	r.t	10 h	80
13	5	H_2_O:EtOH (1:1)	100 °C	1:30 h	75
14	10	H_2_O:EtOH (1:1)	60 °C	30 min	82
15	20	H_2_O:EtOH (1:1)	60 °C	30 min	87
16	30	H_2_O:EtOH (1:1)	60 °C	25 min	89
17	40	H_2_O:EtOH (1:1)	60 °C	20 min	93
18	50	H_2_O:EtOH (1:1)	60 °C	20 min	94

The reaction was carried out at different temperatures also (Table 1, entry 10–13), ranging from r.t to 100 °C and it was found that at 60 °C the yield of reaction was better than other temperatures and the reaction time was reduced to 45 min.

The influence of amount of catalyst on the yield and time was also investigated (Table 1, entry 14–18). By increasing the amount of catalyst from 5 to 40 mg, reaction efficiency increased by 93% and reaction time was decreased to 20 min. Further increase in catalyst amount had no profound effect on the yield of the desired product. Based on the above results, the optimal conditions were established to be the use of 30 mg γFe_2_O_3_@Sh@Cu_2_O as the catalyst in H_2_O:EtOH (1:1) at 60 °C. Some nano materials such as nano Fe_3_O_4_, CuFe_2_O_4_ with sodium ascorbate, Humic acid (HA), Fe_3_O_4_@HA, Fe_3_O_4_@HA@Cu, Sh, Fe_3_O_4_@Sh and some copper salt such as CuBr_2_ were test in optimum condition. However, in most cases, the reaction efficiency was not improved. A clear improvement of the yield was observed when Fe_3_O_4_@HA@Cu was added, which was foreseeable because it was the substance of the Sh, however the time is still longer than satisfactory (Table 2, entry 7).

**Table 2:** Screening catalysts for the three-component reaction of benzyl bromide, sodium azide and phenyl acetylene

Entry	Catalyst (30 mg)	Time	Isolated yield
1	Fe_3_O_4_	5 h	–
2	Sh	5 h	–
3	Fe_3_O_4_@Sh	5 h	–
4	γFe_2_O_3_@Sh@Cu_2_O	20 min	94
5	HA	5 h	Trace
6	Fe_3_O_4_@HA	5 h	15
7	Fe_3_O_4_@HA@Cu	45 min	90
8	Cu_2_Fe_2_O_4_, sodium ascorbate	5 h	15
9	CuBr_2_	5 h	Trace

Experimental conditions: benzyl bromide (1.3 mmol), phenyl acetylene (1.0 mmol), sodium azide (1.3 mmol), catalyst (30 mg), solvent (H_2_O:EtOH 2 mL), 60 °C

**Fig. 15:**
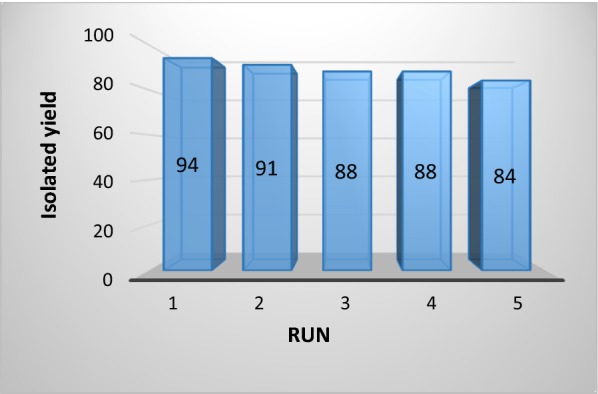
Reusability of γFe_2_O_3_@Sh@Cu_2_O in the model reaction

In practice the effortlessness separation and recyclability are crucial factors for a heterogeneous catalyst. To evaluate the effectiveness of γFe_2_O_3_@Sh@Cu_2_O, its recyclability was verified in the model reaction. After completion of the reaction, the catalyst was recovered by an external magnet and washed several times with EtOH, and then re-used after drying it at 60 °C. The recycled catalyst was used 5 times more, with little change in the efficiency and reaction time (Fig. 15).

**Fig. 16:**
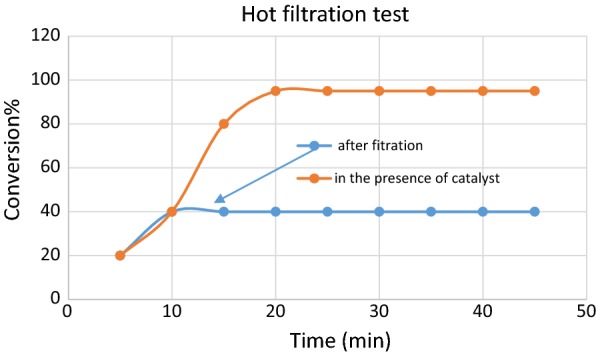
Hot filtration test to investigate heterogeneous nature of catalyst

The catalyst leaching study was performed to determine the heterogeneity of the solid catalyst. The catalytically active particles were removed from the reaction by filtration after 10 min using a hot frit. A reaction monitoring and metals measurement in solution indicated that there is practically no copper leaching during the reaction and the reaction rate decreased significantly after hot filtration (Fig. 16).

We began to make derivatives of this three-component reaction with the optimal reaction conditions we have in place. Different benzyl halides were explored under optimal conditions and the corresponding triazoles were obtained in good to excellent yields (Table 3). The reaction of terminal aryl alkyne bearing electron-donating or electron-withdrawing groups with benzyl halides and sodium azide leads to the corresponding products with high regioselectivity and yields. Satisfactorily, aryl alkyne having electron-donating substituents worked well and delivered expected products in high to excellent yields.

**Table 3:** Scope of reaction of benzyl halides with alkynes and sodium azide catalyzed by magnetic γFe_2_O_3_@Sh@Cu_2_O

Entry	R^1^	R^2^	Product	Isolated yield %	Time (min)	TON	TOF (h^−1^)	M.p [refs.]
1	H	H	**4a**	93	20	3.5 * 10^5^	1.0 * 10^6^	125–127 [9]
2	H	4-Me	**4b**	77	20	2.9 * 10^5^	8.8 * 10^5^	147–148 [44]
3	H	4-OMe	**4c**	97	25	3.7 * 10^5^	8.9 * 10^5^	138–140 [20]
4	4-Br	H	**4d**	98	20	3.7 * 10^5^	7.5 * 10^5^	147–149 [22]
5	4-Br	4-Me	**4e**	72	20	2.7 * 10^5^	8.3 * 10^5^	165–167 [45]
6	4-Br	4-OMe	**4f**	98	20	3.7 * 10^5^	1.1 * 10^6^	164–166 [24]
7	4-OMe	H	**4g**	73	20	2.8 * 10^5^	8.4 * 10^5^	125–127 [46]
8	4-OMe	4-Me	**4h**	57	20	2.1 * 10^5^	8.2 * 10^5^	132–135 [20]
9	4-OMe	4-OMe	**4i**	66	20	2.5 * 10^5^	7.6 * 10^5^	98–100 [47]
10	4-NO_2_	H	**4j**	53	25	2.0 * 10^5^	6.1 * 10^5^	152–154 [48]
11	4-NO_2_	4-Me	**4k**	50	25	1.9 * 10^5^	5.7 * 10^5^	242–245 [49]
12	4-NO_2_	4-OMe	**4l**	70	25	2.6 * 10^5^	6.4 * 10^5^	123.5–125.5 [50]
13	2-Cl	H	**4m**	88	20	3.3 * 10^5^	8.1 * 10^5^	84–86 [9]
14	2-Cl	4-Me	**4n**	88	20	3.3 * 10^5^	8.1 * 10^5^	117–118 [23]
15	2-Cl	4-OMe	**4o**	75	20	2.8 * 10^5^	3.4 * 10^5^	137–138^a^

^a^New product

**Fig. 17:**
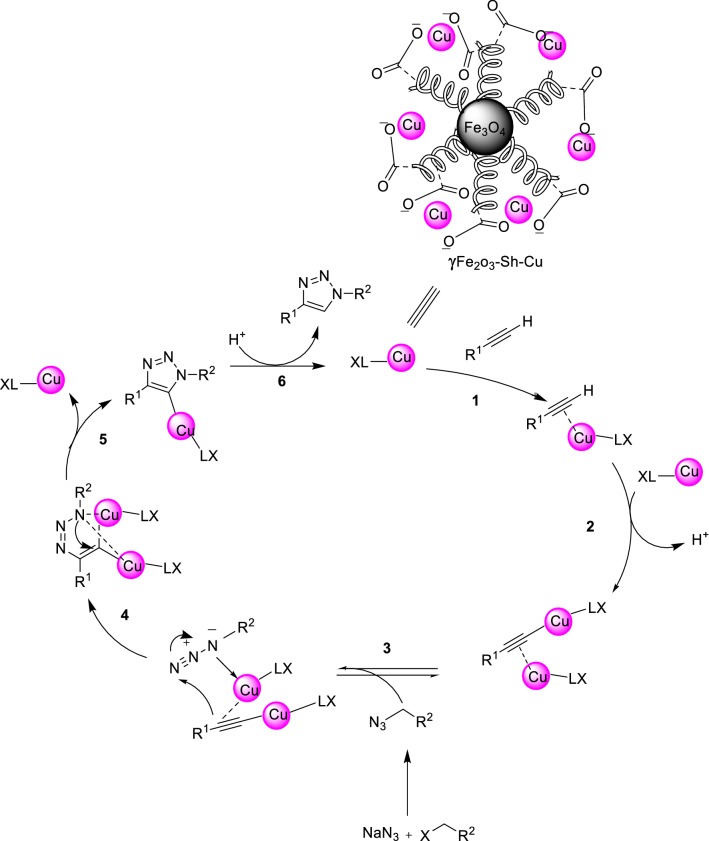
Conceivable catalytic pathway of the copper-catalyzed azide-alkyne cycloaddition (CuAAC)

Next, the reactivity of various benzyl halides were evaluated. It has been observed that reaction does not occur well when we use benzyl halides with electron-withdrawing substituents. Therefore, it is predicted that the first part of the reaction, which is the formation of benzyl azide, proceeds through the SN1 mechanism, while the second part of the reaction, namely the formation of the triazole ring, proceeds through an interesting pathway. The probable reason may be that in benzyl halides the positive charge at benzylic position is stabilized due to conjugation with the phenyl ring, on the other hand, sodium azide is a weak nucleophile, therefore, the suggested pathway is SN1. Coordination of Cu(I) to the alkyne is slightly endothermic in MeCN, but exothermic in water, which is in agreement with an observed rate acceleration in water. However, coordination of Cu to the acetylene does not accelerate a 1,3-dipolar cycloaddition. Such a process has been calculated to be even less favorable than the uncatalyzed 1,3-dipolar cycloaddition. Instead, an σ-bound copper acetylide bearing a π-bound copper coordinates the azide. Then, an unusual six-membered copper metallacycle is formed. The 2nd copper atom acts as a stabilizing donor ligand. Ring contraction to a triazolyl-copper derivative is followed by protonolysis that delivers the triazole product and closes the catalytic cycle [43] (Fig. 17). The final product of this three-component reaction here is 1,4-diaryl-1,2,3-triazole. These results have successfully demonstrated that this catalyst can easily be used for the synthesis of the click synthesis of 1,4-disubstiuted-1,2,3-triazoles.

To compare the catalytic activity of the synthesized catalyst with other reported heterogeneous catalysts for the three-component reaction of benzyl bromide, sodium azide and phenyl acetylene, the TON and TOF are calculated and tabulated in Table 4. As it can be perceived, γFe_2_O_3_@Sh@Cu_2_O shows higher TON and TOF (entry 7, Table 4).

**Table 4:** Comparison of three-component reaction of benzyl bromide, sodium azide and phenyl acetylene under different condition using different catalysts

Entry	Catalyst and catalyst loading	Solvent	Condition	Time (min)	Yield (%)	TON	TOF	Refs.
1	NiFe_2_O_4_-glutamate-Cu (1 mol%)	H_2_O	r.t	240	92	9.2 * 10^3^	2.3 * 10^3^	[5]
2	CuNPs@agarose (0.05 mol%)	H_2_O	40 °C	480	96	1.9 * 10^5^	2.4 * 10^4^	[7]
3	OSPs-CuBr^a^ (1 mol%)	H_2_O	70 °C	240	91	9.1 * 10^3^	2.2 * 10^3^	[8]
4	Cu/c^b^ (5 mol%)	H_2_O	100 °C	40	92	1.8 * 10^3^	2.8 * 10^3^	[9]
5	Cu cat^c^ (0.25 mol%) Sodium ascorbate (10 mol%)	t-BuOH: H_2_O (1:3)	50 °C	90	99	3.9 * 10^4^	2.6 * 10^4^	[17]
6	MNPs@FGly^d^ (0.5 mol%) Sodium ascorbate	t-BuOH: H2O	60 °C	240	99	1.9 * 10^4^	4.9 * 10^3^	[19]
7	Cu/SD^e^ (6 mol%)	H_2_O	Sonication	15	95	1.6 * 10^3^	6.3 * 10^3^	[51]
8	γFe_2_O_3_@Sh@Cu_2_O (0.025 mol%)	H_2_O:EtOH (1:1)	60 °C	20	93	3.7 * 10^5^	1.0 * 10^6^	This work

^a^Oyster shell powders

^b^Cu(I) on charcoal

^c^Self-assembled polymeric imidazole-copper catalyst

^d^Fe_3_O_4_-silicacoated@functionalized 3-glycidoxypropyltrimethoxysilane

^e^Copper/sandarac

## Conclusion

In summary, a recyclable hybrid magnetic mesoporous material γFe_2_O_3_@Sh@Cu_2_O was developed by click reaction between Sh decorated Fe_3_O_4_ and copper acetate. The analysis revealed that during coating of Fe_3_O_4_@Sh using copper salt (II), synchronized redox sorption of Cu^II^ to Cu^I^ occurs at the same time as the oxidation of Fe_3_O_4_ to γFe_2_O_3_.

γFe_2_O_3_ @Sh@Cu_2_O exhibited outstanding catalytic activity for regioselective synthesis of 1,4-disubstituated-1,2,3-triazoles via one pot three-component click reaction of sodium azide, terminal alkynes and benzyl halides in the absence of any reducing agent and base. Mild reaction condition, high yields, high TON and TOF, easy separation of the catalyst using an external magnet, efficient recyclability, and Group-Assisted Purification (GAP) avoiding column chromatography or recrystallization are the merits of this catalytic process.

## Methods

### Materials

All reagents and materials were purchased from commercial sources and used without purification. All of them were analytical grade. ^1^H, ^13^C NMR spectra were recorded on a Bruker Avance DPX 300. The chemical shifts (δ) are given in parts per million and referenced to TMS internal standard. IR spectra were recorded in KBr on Shimadzu FT-IR spectrometer and are reported in wave numbers (cm^−1^). All melting points were measured on a capillary melting point apparatus. All sonication processes were performed using a 400-W probe-type ultrasonic homogenizer from Topsonic Company. Scanning electron microscopy (SEM) was recorded on a VEG//TESCAN 100EM10C-KV, and energy dispersive X-ray spectroscopy (EDX) was recorded on a VEG//TESCAN-XMU. Powder X-ray diffraction (PANalytical X’Pert Pro X-ray diffractometer with the Cu Kɑ), Fourier transform infrared spectroscopy.

### Experimental section

#### Preparation of Fe_3_O_4_ magnetic nanoparticles

Magnetic Fe_3_O_4_ was prepared by precipitation method. A mixed solution of ferrous and ferric ions in the molar ratio 1:2 was prepared by dissolving 2.0 g FeCl_2_·4H_2_O (0.01 mmol) and 5.20 g FeCl_3_·6H_2_O (0.02 mmol) in a round-bottom flask with two openings containing 50 mL H_2_O. This solution stirrer at room temperature for about 15 min to achieve homogeneity solution, when the homogeneous solution was formed, the temperature was elevated to 70 °C. Under reflux, nitrogen gas and stirring conditions and at 70 °C, the ammonia liquid (about 12 mL) was added dropwise over 1 h until the solution became completely black. The solution was allowed to stirrer under basic conditions for another 45 min. Eventually, the obtained precipitated nanoparticles were separated magnetically, washed with water and EtOH until the pH reached 7, and dried at 60 °C for 2 h.

#### Surface modification of nano-Fe_3_O_4_

First, 0.1 g of Sh powder was dispersed in EtOH (10 mL) and sonicated for 1 h at room temperature (solution A). Secondly, a suspension of Fe_3_O_4_ nanoparticles (0.2 g, 0.86 mmol) in 15 mL EtOH was sonicated for 30 min at room temperature (solution B). Glutaraldehyde (1 mL, 10.6 mmol), as a linker, was then added to the solution B and the mixture was subjected to additional sonication for 30 min at room temperature. Thirdly, solutions A and B are mixed and sonicated for 2 h at room temperature. Finally, the obtained precipitate Fe_3_O_4_@Sh, was magnetically separated, washed several times with EtOH and dried at 60 °C for 12 h.

#### Immobilization of Cu on Fe_3_O_4_@Sh

0.4 g of the prepared Fe_3_O_4_@Sh was magnetically stirred under reflux condition in EtOH (30 mL) until the obtention of a homogeneous solution. A solution of CuBr_2_ (0.4 g, 0.002 mol) in EtOH (5 mL) was added drop wise to the reaction mixture and mixture stirred for 2 h. Eventually, the catalysts harvested with the aid of a magnet, washed with EtOH Several times and dried at 60 °C for 12 h.

#### General procedure for synthesis of 1,2,3-triazoles in water:EtOH (1:1)

NaN_3_ (1.3 mmol), alkyne (1 mmol) and benzyl halide (1.3 mmol) were added to a suspension of γFe_2_O_3_@Sh@Cu_2_O (0.025 mol% Cu, 0.04 g γFe_2_O_3_@Sh@Cu_2_O) in H_2_O:EtOH (1:1) (2 mL). The reaction mixture was stirred at 60 °C and monitored by TLC. After completion of the reaction, the catalyst was easily removed from reaction mixture using an external magnet. Then solvent was evaporated with heat and needle-shaped crystals were formed. Finally, crystal products washed with water and normal hexane several times and dried at 60 °C for 6 h.

#### Leaching test

To determine the copper leakage from the catalyst during the reaction, leaching test was performed hot filtration test for click reaction of benzyl halide **1**, phenylacetylene **3** and Sodium azide. The catalytically active particles were removed from the reaction by filtration after 10 min using a hot frit. After hot filtration, the yield of the reaction no longer changes and stagnates at around 40%.

#### Characterization data

1-(4-Bromobenzyl)-4-(4-methoxyphenyl)-1,2,3-triazole (**4f**). White solid; IR (KBr): 3087, 3043, 3010, 2956, 2929, 2900, 2831, 1612, 1558, 1492, 1454, 1350, 1298, 1249, 1078, 1029, 821, 761, 524, 476 cm^−1^. ^1^H NMR (DMSO, 300 MHZ) δ = 3.774 (s, 3H), δ = 5.619 (s, 2H), δ = 7.013 (d, J = .028, 2H), δ = 7.320 (d, J = 0.027, 2H), δ = 7.599 (d, J = 0.027, 2H),  δ  = 7.782 (d, J = .027, 2H), δ = 8.527 (s, 1H) ppm; ^13^C NMR (CDCl_3_, 75 MHZ) δ = 52.724, 55.629, 114.781, 121.079, 121.867, 123.718, 127.028, 130.606, 132.162, 135.897, 147.130, 159,546 ppm.

1-(2-Chlorobenzyl)-4-(4-methoxyphenyl)-1*H*-1,2,3-triazole (**4o**). White solid; IR (KBr): 3113, 3107, 2997, 2933, 2835, 1614, 1560, 1498, 1448, 1249, 1174, 1035, 829, 752, 700, 609, 526 cm^−1^. ^1^H NMR (DMSO, 300 MHZ) δ = 3.382 (s, 3H), δ = 3.784 (s, 3H), δ = 5.738 (s, 2H), δ = 6.996 (d, J = 0.02, 2H), δ = 7.283 (t, j = 0.11, 3H), δ = 7.784 (d, J = 0.02, 2H), δ = 8.499 (s, 1H); ^13^C NMR (CDCl_3_, 75 MHZ) δ = 51.193, 55.602, 114.752, 121.379, 123.657, 125.411, 127.052, 128.220, 130.089, 130.679, 130.918, 133.085,133.716, 146.921, 159.526 ppm.

1-(2-Chlorobenzyl)-4-(*p*-tolyl)-1*H*-1,2,3-triazole (**4n**). White solid; IR (KBr): 3124, 3060, 2970, 2937, 2777, 1654, 1590, 1443, 1425, 1350, 1288, 1220, 1203, 1100, 1082, 1043, 1016, 860, 838, 802, 781, 730, 690, 538 cm^−1^. ^1^HNMR (DMSO, 300 MHZ) δ = 2.313 (s, 3H), δ = 5.743 (s, 2H), δ = 7.228 (m, 3H), δ = 7.351 (m, 2H), δ = 7.5714 (d, J = 0.021, 1H), δ = 7.745 (d, J = 0.026, 2H), δ = 8.557 (s, 1H); ^13^C NMR (CDCl_3_, 75 MHZ) δ = 51.221, 121.882, 125.643, 128.200, 128.320, 129.867, 130.086, 130.680, 130.954, 133.117, 133.668, 137.690, 147.054 ppm.

## Supplementary information

**Additional file 1. Figure S1.** ^1^HNMR of 1-(4-bromobenzyl)-4-(4-methoxyphenyl)-1,2,3-triazole. **Figure S2.** Expanded ^1^HNMR spectra of 1-(4-bromobenzyl)-4-(4-methoxyphenyl)-1,2,3-triazole (aromatic region). **Figure S3.** ^13^CNMR of 1-(4-bromobenzyl)-4-(4-methoxyphenyl)-1,2,3-triazole. **Figure S4.** ^13^CNMR of 1-(4-bromobenzyl)-4-(4-methoxyphenyl)-1,2,3-triazole. **Figure S5.** ^1^HNMR of 1-(2-chlorobenzyl)-4-(4-methoxyphenyl)-1H-1,2,3-triazole. **Figure S6.** Expanded ^1^HNMR spectra of 1-(2-chlorobenzyl)-4-(4-methoxyphenyl)-1*H*-1,2,3-triazole (aromatic region). **Figure S7.** ^13^CNMR of 1-(2-chlorobenzyl)-4-(4-methoxyphenyl)-1*H*-1,2,3-triazole. **Figure S8.** Expanded ^13^CNMR spectra of 1-(2-chlorobenzyl)-4-(4-methoxyphenyl)-1H-1,2,3-triazole. **Figure S9.** ^1^HNMR of 1-(2-chlorobenzyl)-4-(4-*p*-tolyl)-1*H*-1,2,3-triazole. **Figure S10.** Expanded ^1^HNMR spectra of 1-(2-chlorobenzyl)-4-(*p*-tolyl)-1*H*-1,2,3-triazole (aromatic region). **Figure S11.** ^13^CNMR of 1-(2-chlorobenzyl)-4-(*p*-tolyl)-1*H*-1,2,3-triazole. **Figure S12.** Expanded ^13^CNMR spectra of 1-(2-chlorobenzyl**)-**4-(p-tolyl)-1*H*-1,2,3-triazole.

## Data Availability

All data generated or analysed during this study are included in this published article and Additional files.

## Abbreviations

BET: Brunauer–Emmett–Teller
BJH: Barrett–Joyner–Halenda
CuAAC: copper-catalyzed azide-alkyne cycloaddition
EDX: energy-dispersive X-ray spectroscopy
FESEM: field emission scanning electron microscopy
FGly: functionalized 3-glycidoxypropyltrimethoxysilane
FGT: ferrite-glutathione-copper
FTIR: Fourier transform infrared
HA: humic acid
HMOP: hierarchical mesoporous organic polymer
ICP: inductively coupled plasma
MNPs: magnetic nanoparticles
NPs: nanoparticles
OSPs: oyster shell powders
SBA: Santa Barbara Amorphous
SEM: scanning electron microscopy
Sh: Shilajit
TGA: thermal gravimetric analysis
TON: turnover number
TOF: turnover frequency
vsm: vibrating sample magnetometer
XPS: X-ray photoelectron spectroscopy
XRD: X-ray diffraction
