# Supplementary material for: Magnetic γFe2O3@Sh@Cu2O: an efficient solid-phase catalyst for reducing agent and base-free click synthesis of 1,4-disubstituted-1,2,3-triazoles
Source: BMC Chem. 2020 Jan 7;14(1):1. doi: 10.1186/s13065-019-0657-9 (PMC6945398; doi:10.1186/s13065-019-0657-9)
Supplement: Supplementary file 1 — Additional file 1. Figure S1. 1HNMR of 1-(4-bromobenzyl)-4-(4-methoxyphenyl)-1,2,3-triazole. Figure S2. Expanded 1HNMR spectra of 1-(4-bromobenzyl)-4-(4-methoxyphenyl)-1,2,3-triazole (aromatic region). Figure S3. 13CNMR of 1-(4-bromobenzyl)-4-(4-methoxyphenyl)-1,2,3-triazole. Figure S4. 13CNMR of 1-(4-bromobenzyl)-4-(4-methoxyphenyl)-1,2,3-triazole. Figure S5. 1HNMR of 1-(2-chlorobenzyl)-4-(4-methoxyphenyl)-1H-1,2,3-triazole. Figure S6. Expanded 1HNMR spectra of 1-(2-chlorobenzyl)-4-(4-methoxyphenyl)-1H-1,2,3-triazole (aromatic region). Figure S7. 13CNMR of 1-(2-chlorobenzyl)-4-(4-methoxyphenyl)-1H-1,2,3-triazole. Figure S8. Expanded 13CNMR spectra of 1-(2-chlorobenzyl)-4-(4-methoxyphenyl)-1H-1,2,3-triazole. Figure S9. 1HNMR of 1-(2-chlorobenzyl)-4-(4-p-tolyl)-1H-1,2,3-triazole. Figure S10. Expanded 1HNMR spectra of 1-(2-chlorobenzyl)-4-(p-tolyl)-1H-1,2,3-triazole (aromatic region). Figure S11. 13CNMR of 1-(2-chlorobenzyl)-4-(p-tolyl)-1H-1,2,3-triazole. Figure S12. Expanded 13CNMR spectra of 1-(2-chlorobenzyl)-4-(p-tolyl)-1H-1,2,3-triazole. [file 13065_2019_657_MOESM1_ESM.docx]

**Additional file 1**

Synthesis of New Magnetic Hybrid Materials γFe_2_O_3_@Sh@Cu_2_O Based on Natural Shilajit Resin and their Performance as Heterogeneous Catalyst in Reducing Agent and Base-Free Click Synthesis of 1,4-disubstituted 1,2,3-Triazoles

*Fereshteh Norouzi*1, *Shahrzad Javanshir*1*

*[Shjavan@iust.ac.ir](mailto:Shjavan@iust.ac.ir)

**Table of content**

|  | Page |
| --- | --- |
| Fig. S1. ^1^HNMR of 1-(4-bromobenzyl)-4-(4-methoxyphenyl)-1,2,3-triazole | 2 |
| Fig. S2. Expanded ^1^HNMR spectra of 1-(4-bromobenzyl)-4-(4-methoxyphenyl)-1,2,3-triazole (aromatic region) | 2 |
| Fig. S3. ^13^CNMR of 1-(4-bromobenzyl)-4-(4-methoxyphenyl)-1,2,3-triazole | 3 |
| Fig. S4. ^13^CNMR of 1-(4-bromobenzyl)-4-(4-methoxyphenyl)-1,2,3-triazole | 3 |
| Fig. S5. ^1^HNMR of 1-(2-chlorobenzyl)-4-(4-methoxyphenyl)-1H-1,2,3-triazole | 4 |
| Fig. S6. Expanded ^1^HNMR spectra of 1-(2-chlorobenzyl)-4-(4-methoxyphenyl)-1H-1,2,3-triazole (aromatic region) | 4 |
| Fig. S7. ^13^CNMR of 1-(2-chlorobenzyl)-4-(4-methoxyphenyl)-1H-1,2,3-triazole | 5 |
| Fig. S8. Expanded ^13^CNMR spectra of 1-(2-chlorobenzyl)-4-(4-methoxyphenyl)-1H-1,2,3-triazole | 5 |
| Fig. S9. ^1^HNMR of 1-(2-chlorobenzyl)-4-(4-p-tolyl)-1H-1,2,3-triazole | 6 |
| Fig. S10. Expanded ^1^HNMR spectra of 1-(2-chlorobenzyl)-4-(p-tolyl)-1H-1,2,3-triazole (aromatic region) | 6 |
| Fig. S11. ^13^CNMR of 1-(2-chlorobenzyl)-4-(p-tolyl)-1H-1,2,3-triazole | 7 |
| Fig. S12. Expanded ^13^CNMR spectra of 1-(2-chlorobenzyl)-4-(p-tolyl)-1H-1,2,3-triazole | 7 |


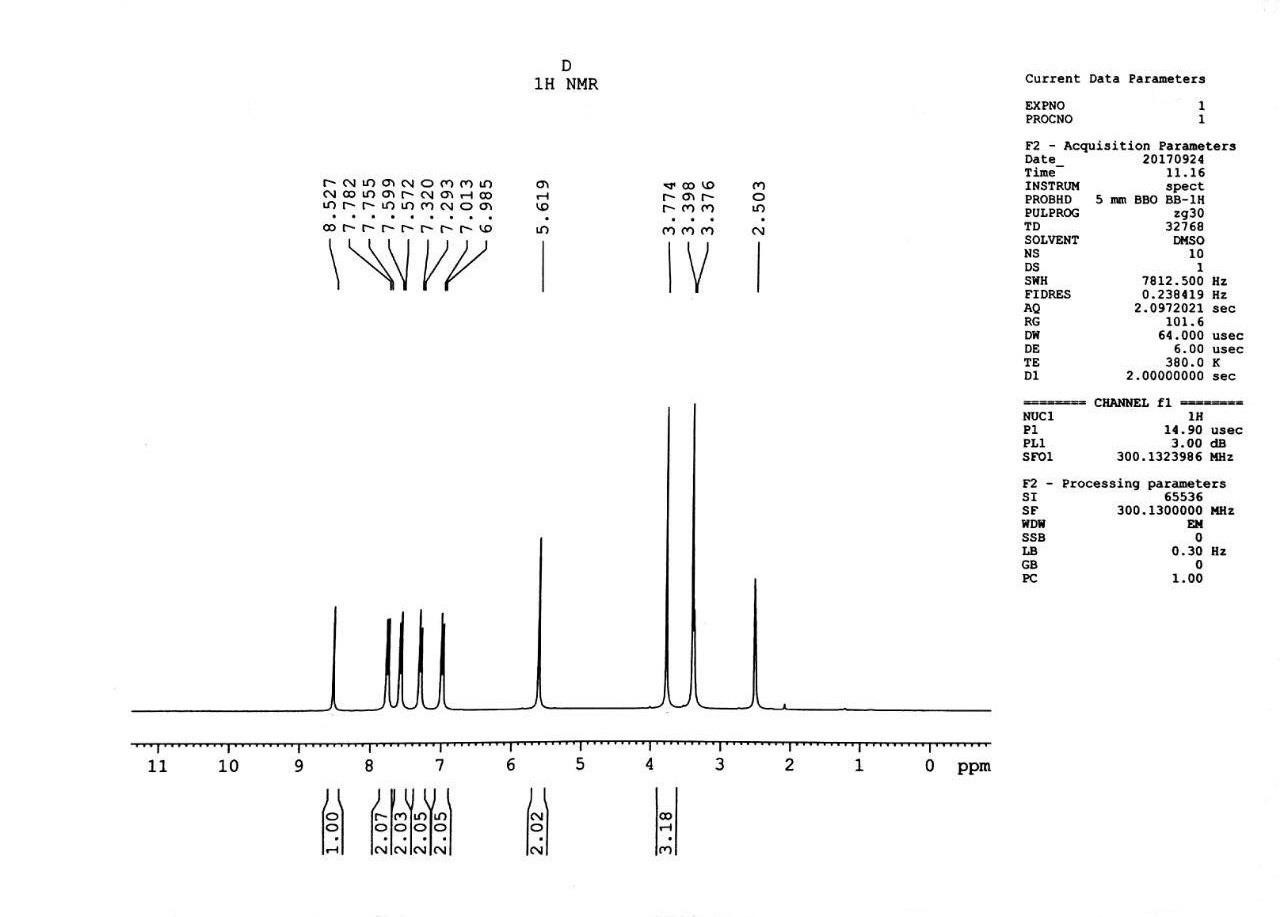


**Fig. S1.** ^1^HNMR of 1-(4-bromobenzyl)-4-(4-methoxyphenyl)-1,2,3-triazole


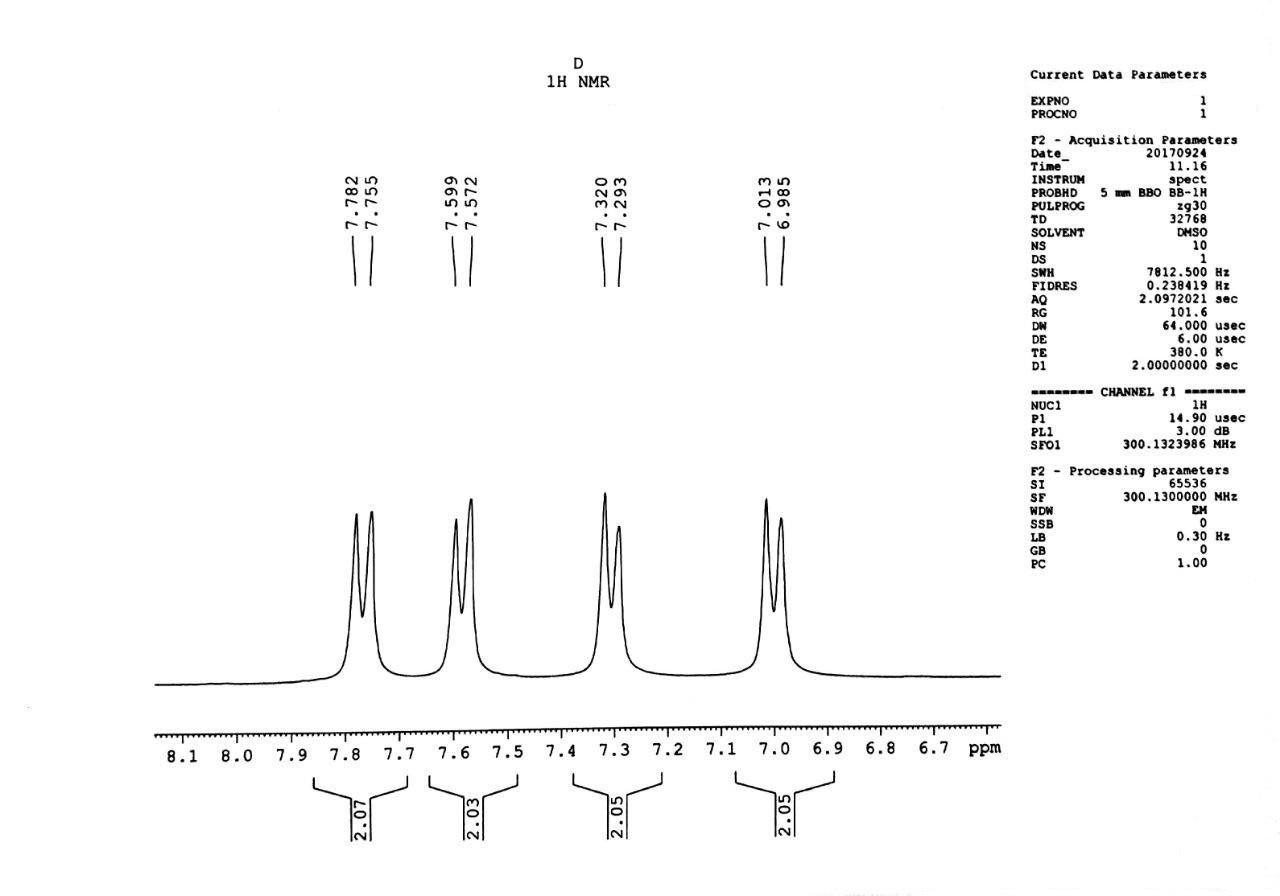


**Fig. S2.** Expanded ^1^HNMR spectra of 1-(4-bromobenzyl)-4-(4-methoxyphenyl)-1,2,3-triazole (aromatic region)


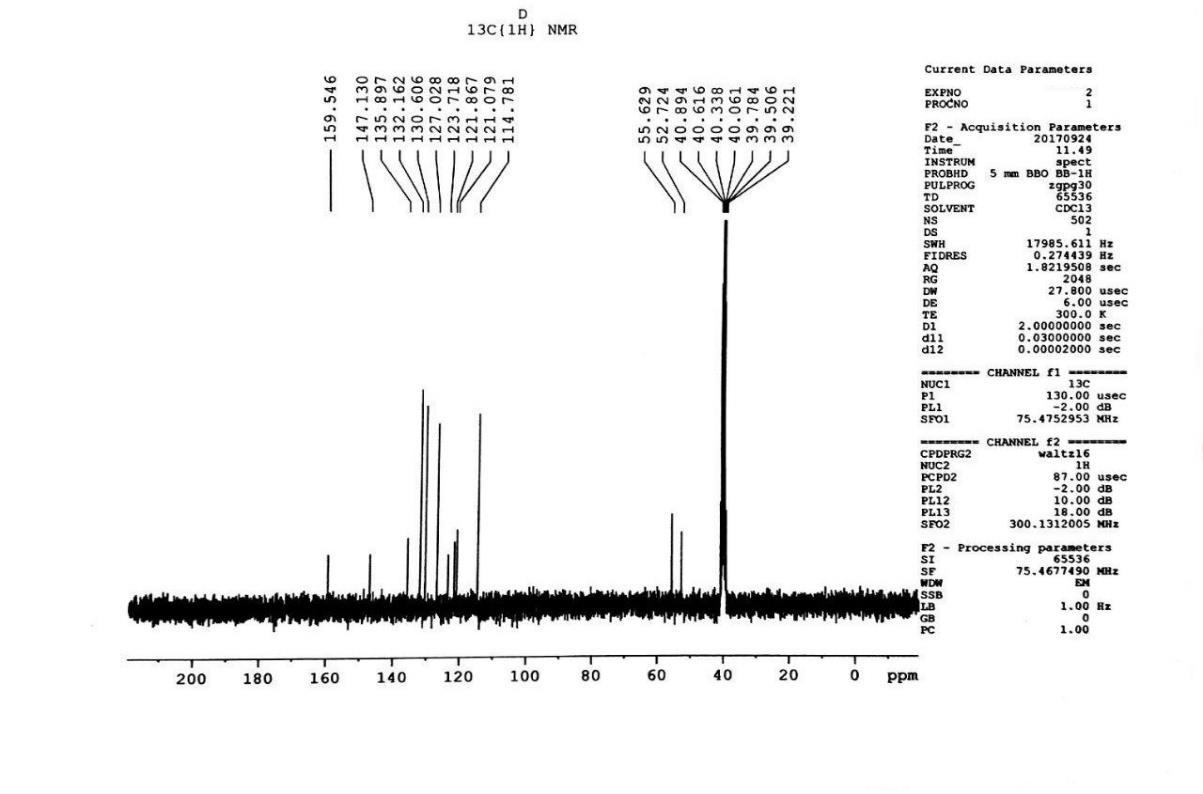


**Fig. S3.** ^13^CNMR of 1-(4-bromobenzyl)-4-(4-methoxyphenyl)-1,2,3-triazole


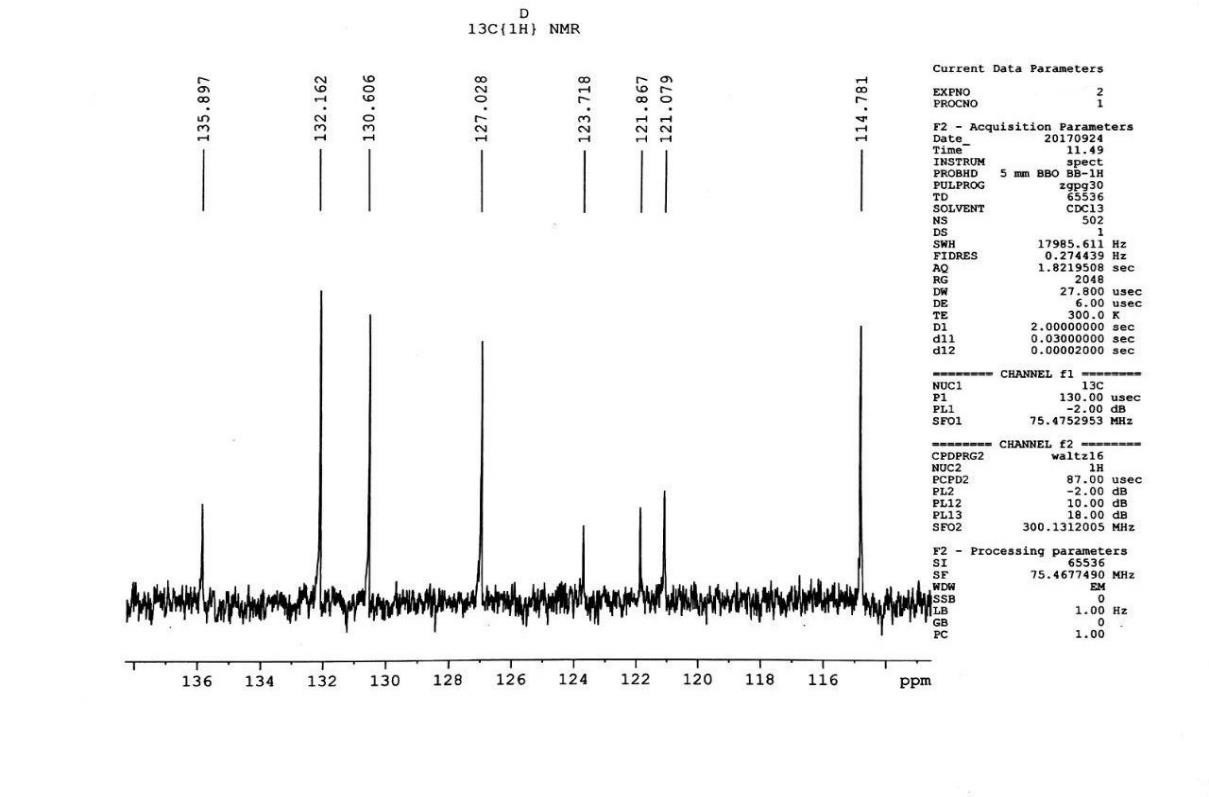


**Fig. S4.** ^13^CNMR of 1-(4-bromobenzyl)-4-(4-methoxyphenyl)-1,2,3-triazole


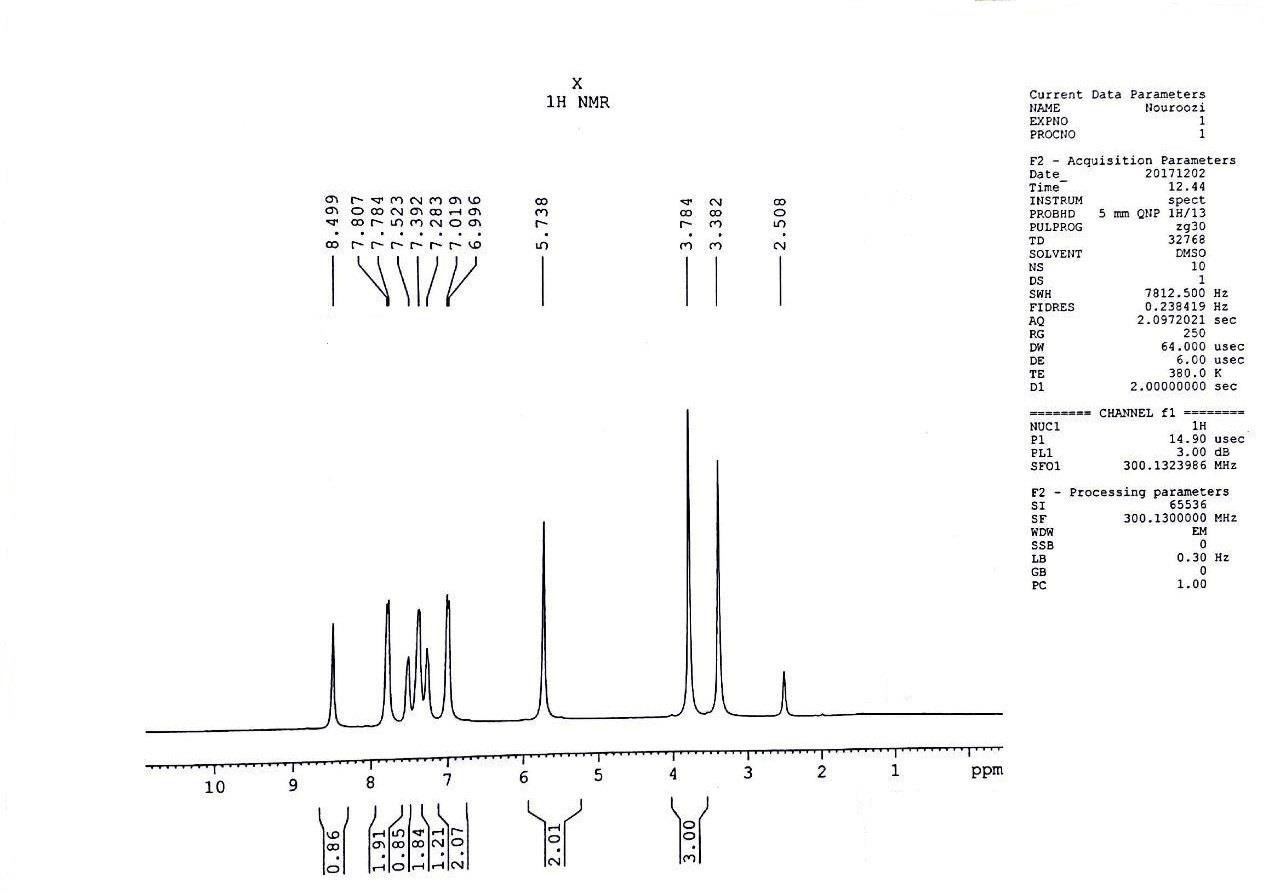


**Fig. S5.** ^1^HNMR of 1-(2-chlorobenzyl)-4-(4-methoxyphenyl)-1H-1,2,3-triazole


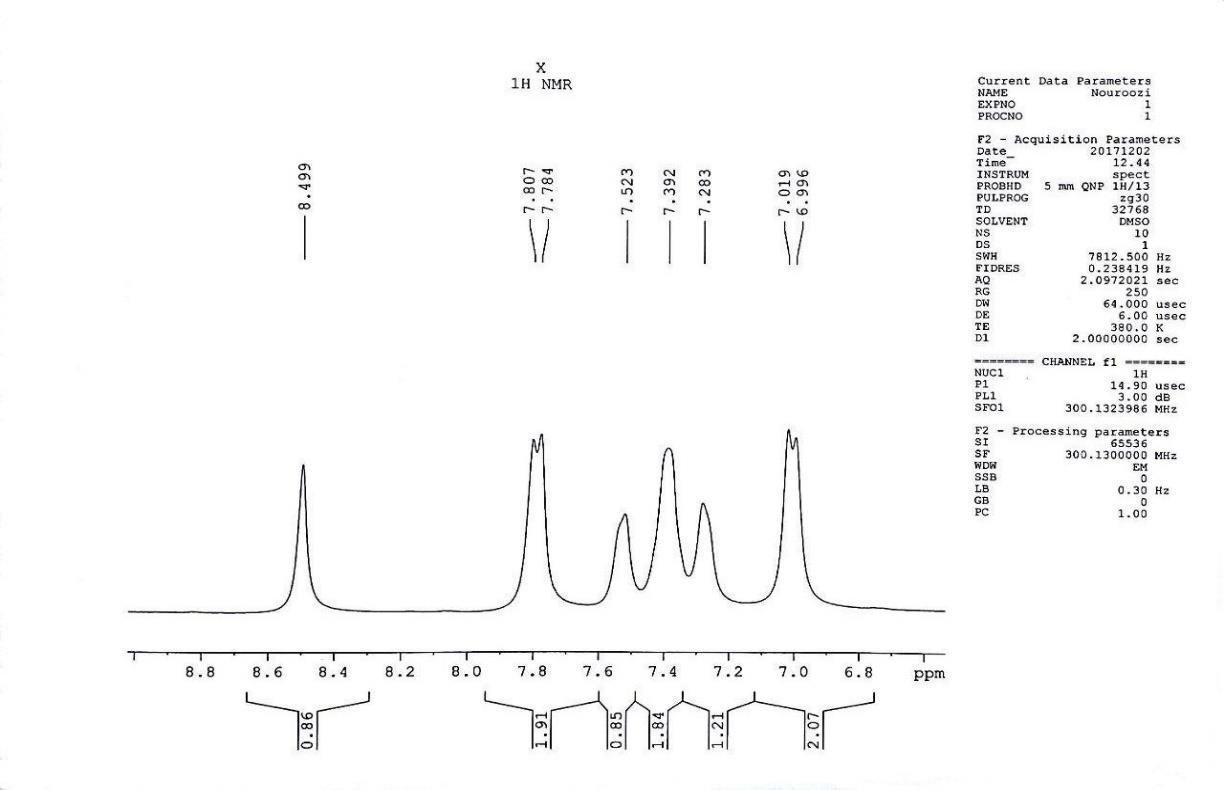


**Fig. S6.** Expanded ^1^HNMR spectra of 1-(2-chlorobenzyl)-4-(4-methoxyphenyl)-1H-1,2,3-triazole (aromatic region)


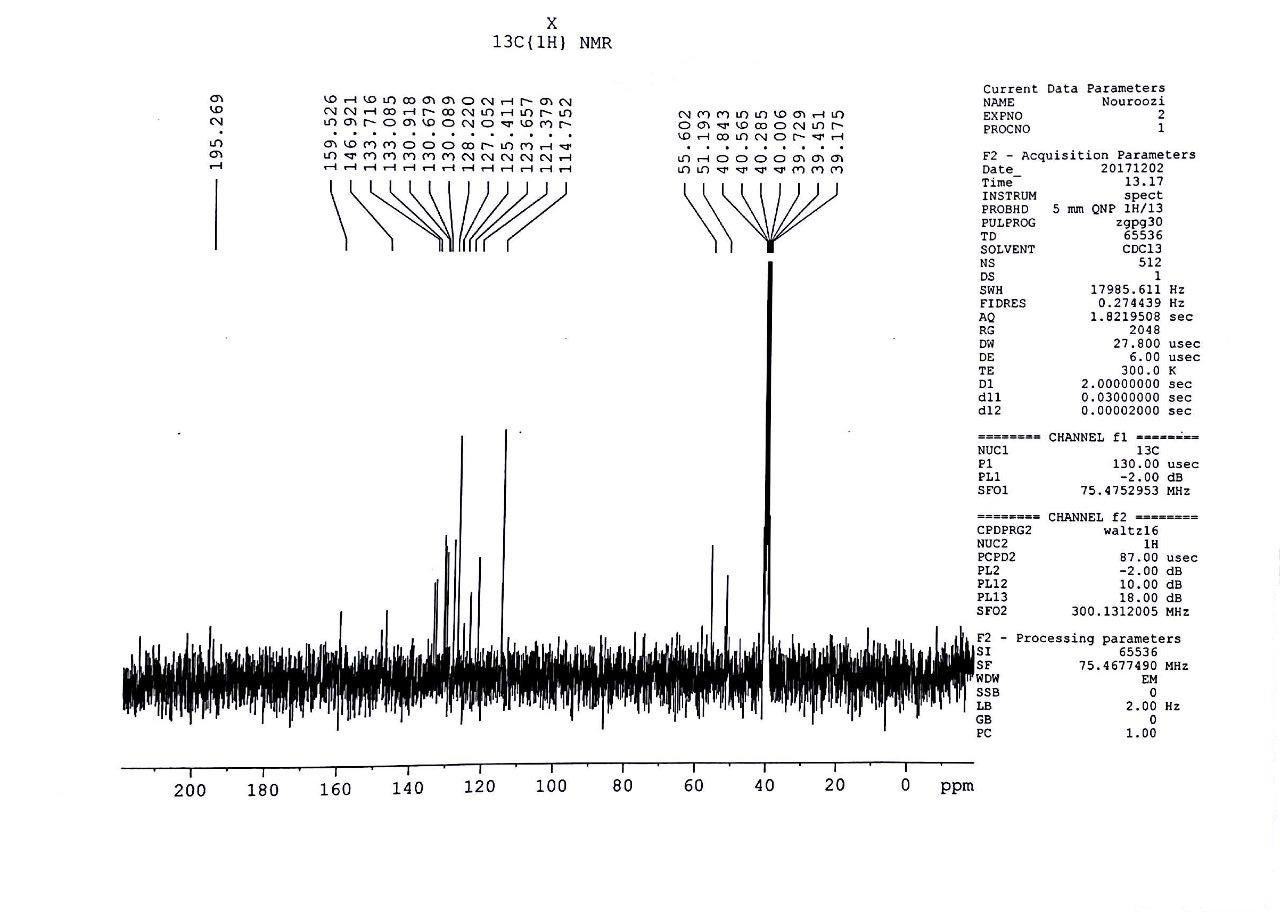


**Fig. S7.** ^13^CNMR of 1-(2-chlorobenzyl)-4-(4-methoxyphenyl)-1H-1,2,3-triazole


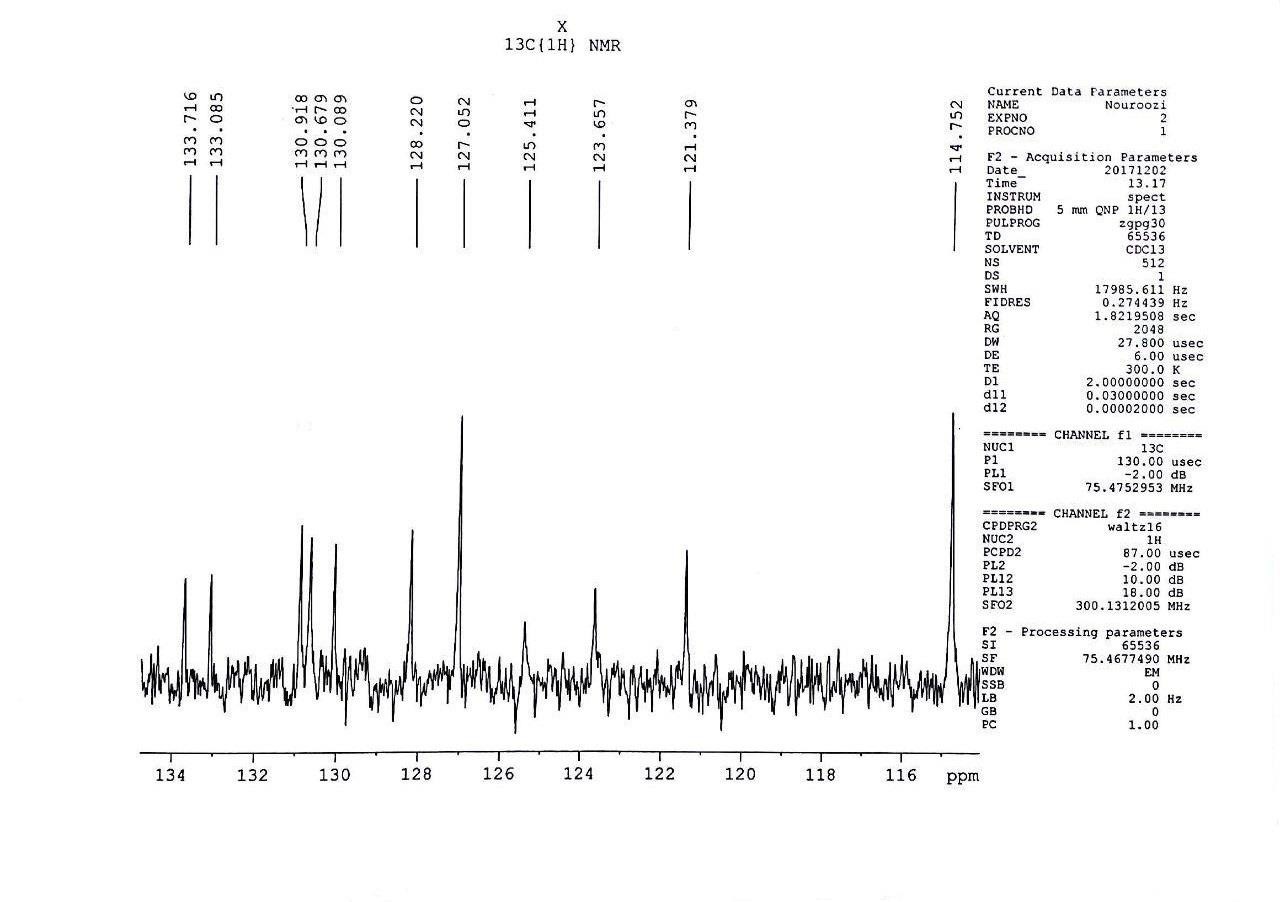


**Fig. S8.** Expanded ^13^CNMR spectra of 1-(2-chlorobenzyl)-4-(4-methoxyphenyl)-1H-1,2,3-triazole.


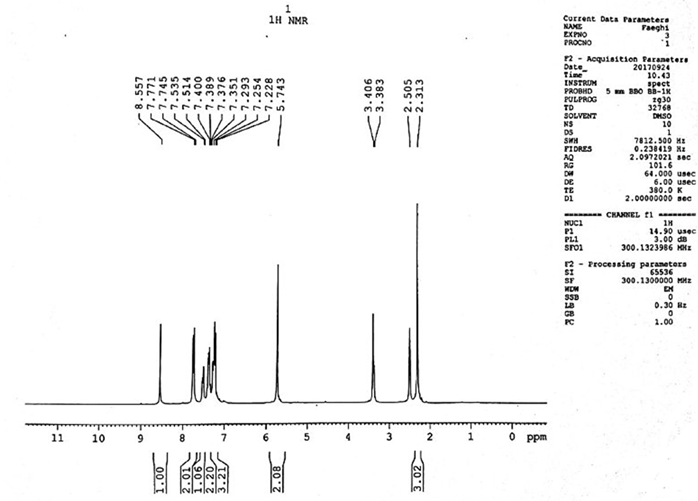


**Fig. S9.** ^1^HNMR of 1-(2-chlorobenzyl)-4-(4-p-tolyl)-1H-1,2,3-triazole


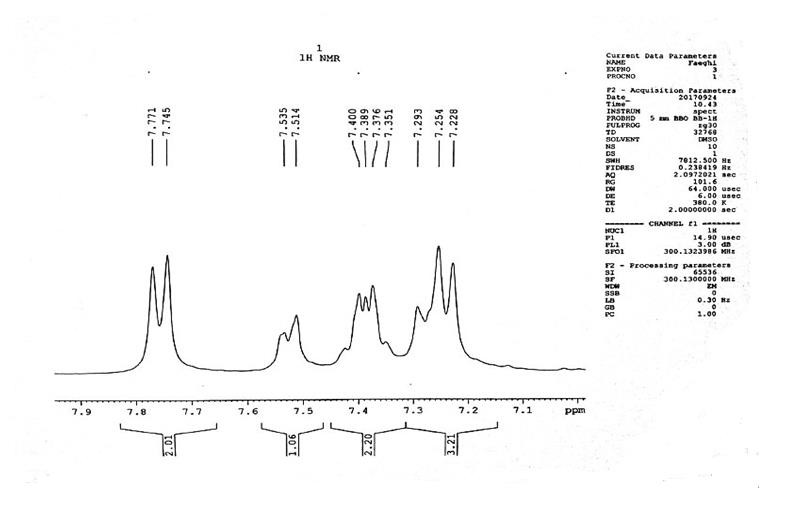


**Fig. S10.** Expanded ^1^HNMR spectra of 1-(2-chlorobenzyl)-4-(p-tolyl)-1H-1,2,3-triazole (aromatic region)


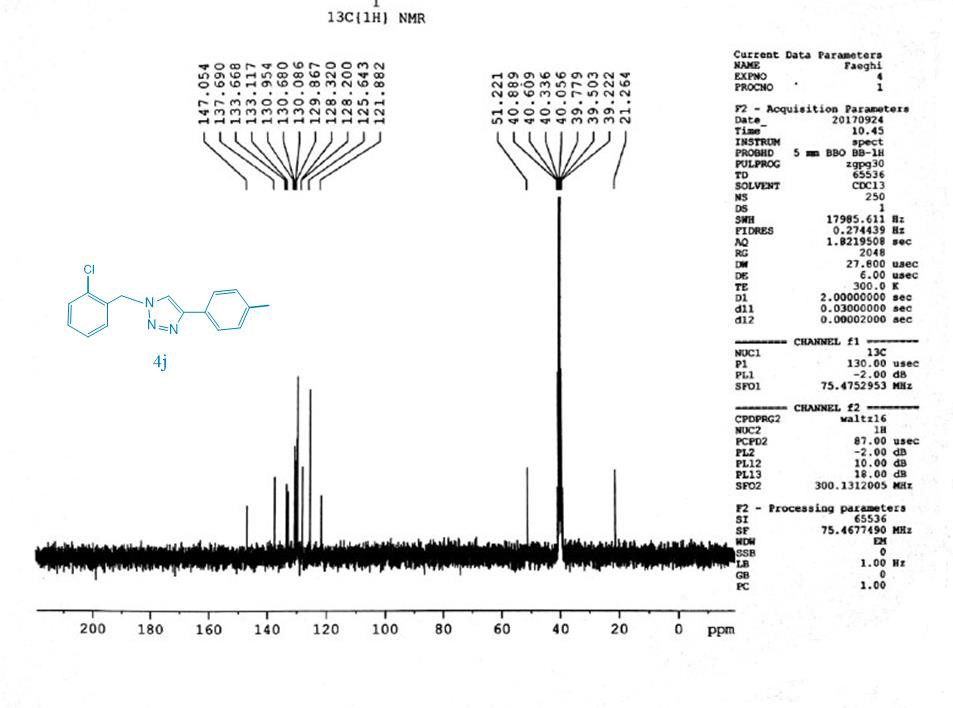


**Fig. S11.** ^13^CNMR of 1-(2-chlorobenzyl)-4-(p-tolyl)-1H-1,2,3-triazole


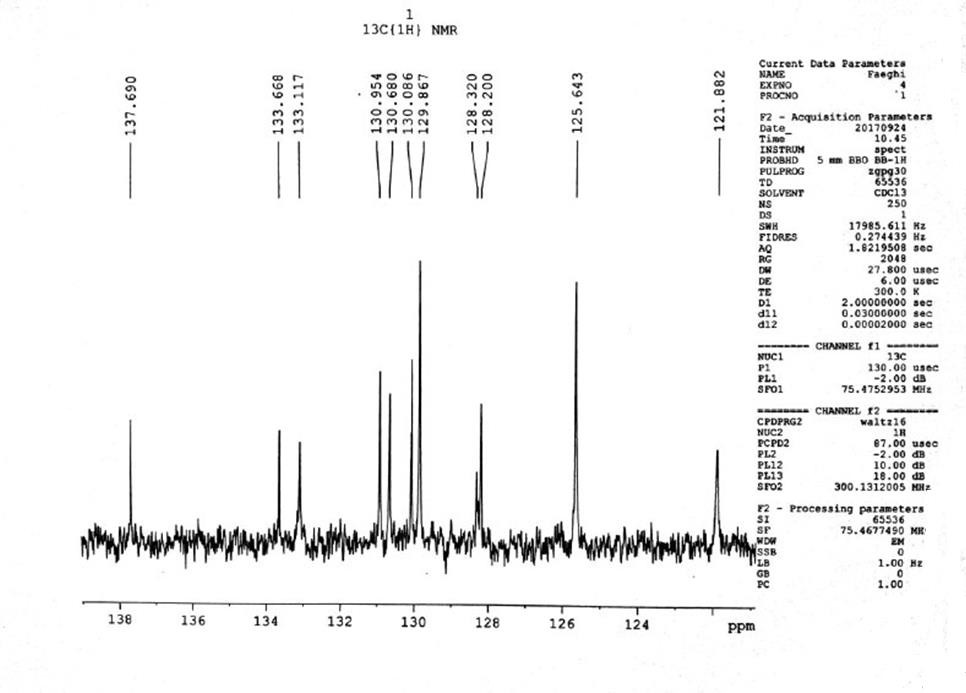


**Fig. S12.** Expanded ^13^CNMR spectra of 1-(2-chlorobenzyl)-4-(p-tolyl)-1H-1,2,3-triazole
